# Measurement of exclusive $\mathrm {\Upsilon }$ photoproduction from protons in $\mathrm {p}$Pb collisions at $\sqrt{\smash [b]{s_{_{\mathrm {NN}}}}} = 5.02\,\text {TeV}$

**DOI:** 10.1140/epjc/s10052-019-6774-8

**Published:** 2019-03-26

**Authors:** A. M. Sirunyan, A. Tumasyan, W. Adam, F. Ambrogi, E. Asilar, T. Bergauer, J. Brandstetter, E. Brondolin, M. Dragicevic, J. Erö, A. Escalante Del Valle, M. Flechl, M. Friedl, R. Frühwirth, V. M. Ghete, J. Grossmann, J. Hrubec, M. Jeitler, A. König, N. Krammer, I. Krätschmer, D. Liko, T. Madlener, I. Mikulec, E. Pree, N. Rad, H. Rohringer, J. Schieck, R. Schöfbeck, M. Spanring, D. Spitzbart, A. Taurok, W. Waltenberger, J. Wittmann, C.-E. Wulz, M. Zarucki, V. Chekhovsky, V. Mossolov, J. Suarez Gonzalez, E. A. De Wolf, D. Di Croce, X. Janssen, J. Lauwers, M. Pieters, M. Van De Klundert, H. Van Haevermaet, P. Van Mechelen, N. Van Remortel, S. Abu Zeid, F. Blekman, J. D’Hondt, I. De Bruyn, J. De Clercq, K. Deroover, G. Flouris, D. Lontkovskyi, S. Lowette, I. Marchesini, S. Moortgat, L. Moreels, Q. Python, K. Skovpen, S. Tavernier, W. Van Doninck, P. Van Mulders, I. Van Parijs, D. Beghin, B. Bilin, H. Brun, B. Clerbaux, G. De Lentdecker, H. Delannoy, B. Dorney, G. Fasanella, L. Favart, R. Goldouzian, A. Grebenyuk, A. K. Kalsi, T. Lenzi, J. Luetic, T. Seva, E. Starling, C. Vander Velde, P. Vanlaer, D. Vannerom, R. Yonamine, T. Cornelis, D. Dobur, A. Fagot, M. Gul, I. Khvastunov, D. Poyraz, C. Roskas, D. Trocino, M. Tytgat, W. Verbeke, B. Vermassen, M. Vit, N. Zaganidis, H. Bakhshiansohi, O. Bondu, S. Brochet, G. Bruno, C. Caputo, A. Caudron, P. David, S. De Visscher, C. Delaere, M. Delcourt, B. Francois, A. Giammanco, G. Krintiras, V. Lemaitre, A. Magitteri, A. Mertens, M. Musich, K. Piotrzkowski, L. Quertenmont, A. Saggio, M. Vidal Marono, S. Wertz, J. Zobec, W. L. Aldá Júnior, F. L. Alves, G. A. Alves, L. Brito, G. Correia Silva, C. Hensel, A. Moraes, M. E. Pol, P. Rebello Teles, E. Belchior Batista Das Chagas, W. Carvalho, J. Chinellato, E. Coelho, E. M. Da Costa, G. G. Da Silveira, D. De Jesus Damiao, S. Fonseca De Souza, H. Malbouisson, M. Medina Jaime, M. Melo De Almeida, C. Mora Herrera, L. Mundim, H. Nogima, L. J. Sanchez Rosas, A. Santoro, A. Sznajder, M. Thiel, E. J. Tonelli Manganote, F. Torres Da Silva De Araujo, A. Vilela Pereira, S. Ahuja, C. A. Bernardes, L. Calligaris, T. R. Fernandez Perez Tomei, E. M. Gregores, P. G. Mercadante, S. F. Novaes, Sandra S. Padula, D. Romero Abad, J. C. Ruiz Vargas, A. Aleksandrov, R. Hadjiiska, P. Iaydjiev, A. Marinov, M. Misheva, M. Rodozov, M. Shopova, G. Sultanov, A. Dimitrov, L. Litov, B. Pavlov, P. Petkov, W. Fang, X. Gao, L. Yuan, M. Ahmad, J. G. Bian, G. M. Chen, H. S. Chen, M. Chen, Y. Chen, C. H. Jiang, D. Leggat, H. Liao, Z. Liu, F. Romeo, S. M. Shaheen, A. Spiezia, J. Tao, C. Wang, Z. Wang, E. Yazgan, H. Zhang, J. Zhao, Y. Ban, G. Chen, J. Li, Q. Li, S. Liu, Y. Mao, S. J. Qian, D. Wang, Z. Xu, Y. Wang, C. Avila, A. Cabrera, C. A. Carrillo Montoya, L. F. Chaparro Sierra, C. Florez, C. F. González Hernández, M. A. Segura Delgado, B. Courbon, N. Godinovic, D. Lelas, I. Puljak, P. M. Ribeiro Cipriano, T. Sculac, Z. Antunovic, M. Kovac, V. Brigljevic, D. Ferencek, K. Kadija, B. Mesic, A. Starodumov, T. Susa, M. W. Ather, A. Attikis, G. Mavromanolakis, J. Mousa, C. Nicolaou, F. Ptochos, P. A. Razis, H. Rykaczewski, M. Finger, M. Finger, E. Carrera Jarrin, S. Khalil, M. A. Mahmoud, Y. Mohammed, S. Bhowmik, R. K. Dewanjee, M. Kadastik, L. Perrini, M. Raidal, C. Veelken, P. Eerola, H. Kirschenmann, J. Pekkanen, M. Voutilainen, J. Havukainen, J. K. Heikkilä, T. Järvinen, V. Karimäki, R. Kinnunen, T. Lampén, K. Lassila-Perini, S. Laurila, S. Lehti, T. Lindén, P. Luukka, T. Mäenpää, H. Siikonen, E. Tuominen, J. Tuominiemi, T. Tuuva, M. Besancon, F. Couderc, M. Dejardin, D. Denegri, J. L. Faure, F. Ferri, S. Ganjour, S. Ghosh, A. Givernaud, P. Gras, G. Hamel de Monchenault, P. Jarry, C. Leloup, E. Locci, M. Machet, J. Malcles, G. Negro, J. Rander, A. Rosowsky, M. Ö. Sahin, M. Titov, A. Abdulsalam, C. Amendola, I. Antropov, S. Baffioni, F. Beaudette, P. Busson, L. Cadamuro, C. Charlot, R. Granier de Cassagnac, M. Jo, I. Kucher, S. Lisniak, A. Lobanov, J. Martin Blanco, M. Nguyen, C. Ochando, G. Ortona, P. Paganini, P. Pigard, R. Salerno, J. B. Sauvan, Y. Sirois, A. G. Stahl Leiton, Y. Yilmaz, A. Zabi, A. Zghiche, J.-L. Agram, J. Andrea, D. Bloch, J.-M. Brom, E. C. Chabert, C. Collard, E. Conte, X. Coubez, F. Drouhin, J.-C. Fontaine, D. Gelé, U. Goerlach, M. Jansová, P. Juillot, A.-C. Le Bihan, N. Tonon, P. Van Hove, S. Gadrat, S. Beauceron, C. Bernet, G. Boudoul, N. Chanon, R. Chierici, D. Contardo, P. Depasse, H. El Mamouni, J. Fay, L. Finco, S. Gascon, M. Gouzevitch, G. Grenier, B. Ille, F. Lagarde, I. B. Laktineh, H. Lattaud, M. Lethuillier, L. Mirabito, A. L. Pequegnot, S. Perries, A. Popov, V. Sordini, M. Vander Donckt, S. Viret, S. Zhang, T. Toriashvili, Z. Tsamalaidze, C. Autermann, L. Feld, M. K. Kiesel, K. Klein, M. Lipinski, M. Preuten, M. P. Rauch, C. Schomakers, J. Schulz, M. Teroerde, B. Wittmer, V. Zhukov, A. Albert, D. Duchardt, M. Endres, M. Erdmann, S. Erdweg, T. Esch, R. Fischer, A. Güth, T. Hebbeker, C. Heidemann, K. Hoepfner, S. Knutzen, M. Merschmeyer, A. Meyer, P. Millet, S. Mukherjee, T. Pook, M. Radziej, H. Reithler, M. Rieger, F. Scheuch, D. Teyssier, S. Thüer, G. Flügge, B. Kargoll, T. Kress, A. Künsken, T. Müller, A. Nehrkorn, A. Nowack, C. Pistone, O. Pooth, A. Stahl, M. Aldaya Martin, T. Arndt, C. Asawatangtrakuldee, K. Beernaert, O. Behnke, U. Behrens, A. Bermúdez Martínez, A. A. Bin Anuar, K. Borras, V. Botta, A. Campbell, P. Connor, C. Contreras-Campana, F. Costanza, V. Danilov, A. De Wit, C. Diez Pardos, D. Domínguez Damiani, G. Eckerlin, D. Eckstein, T. Eichhorn, A. Elwood, E. Eren, E. Gallo, J. Garay Garcia, A. Geiser, J. M. Grados Luyando, A. Grohsjean, P. Gunnellini, M. Guthoff, A. Harb, J. Hauk, M. Hempel, H. Jung, M. Kasemann, J. Keaveney, C. Kleinwort, J. Knolle, I. Korol, D. Krücker, W. Lange, A. Lelek, T. Lenz, K. Lipka, W. Lohmann, R. Mankel, I.-A. Melzer-Pellmann, A. B. Meyer, M. Meyer, M. Missiroli, G. Mittag, J. Mnich, A. Mussgiller, D. Pitzl, A. Raspereza, M. Savitskyi, P. Saxena, R. Shevchenko, N. Stefaniuk, H. Tholen, G. P. Van Onsem, R. Walsh, Y. Wen, K. Wichmann, C. Wissing, O. Zenaiev, R. Aggleton, S. Bein, V. Blobel, M. Centis Vignali, T. Dreyer, E. Garutti, D. Gonzalez, J. Haller, A. Hinzmann, M. Hoffmann, A. Karavdina, G. Kasieczka, R. Klanner, R. Kogler, N. Kovalchuk, S. Kurz, V. Kutzner, J. Lange, D. Marconi, J. Multhaup, M. Niedziela, D. Nowatschin, T. Peiffer, A. Perieanu, A. Reimers, C. Scharf, P. Schleper, A. Schmidt, S. Schumann, J. Schwandt, J. Sonneveld, H. Stadie, G. Steinbrück, F. M. Stober, M. Stöver, D. Troendle, E. Usai, A. Vanhoefer, B. Vormwald, M. Akbiyik, C. Barth, M. Baselga, S. Baur, E. Butz, R. Caspart, T. Chwalek, F. Colombo, W. De Boer, A. Dierlamm, N. Faltermann, B. Freund, R. Friese, M. Giffels, M. A. Harrendorf, F. Hartmann, S. M. Heindl, U. Husemann, F. Kassel, S. Kudella, H. Mildner, M. U. Mozer, Th. Müller, M. Plagge, G. Quast, K. Rabbertz, M. Schröder, I. Shvetsov, G. Sieber, H. J. Simonis, R. Ulrich, S. Wayand, M. Weber, T. Weiler, S. Williamson, C. Wöhrmann, R. Wolf, G. Anagnostou, G. Daskalakis, T. Geralis, A. Kyriakis, D. Loukas, I. Topsis-Giotis, G. Karathanasis, S. Kesisoglou, A. Panagiotou, N. Saoulidou, E. Tziaferi, K. Kousouris, I. Papakrivopoulos, I. Evangelou, C. Foudas, P. Gianneios, P. Katsoulis, P. Kokkas, S. Mallios, N. Manthos, I. Papadopoulos, E. Paradas, J. Strologas, F. A. Triantis, D. Tsitsonis, M. Csanad, N. Filipovic, G. Pasztor, O. Surányi, G. I. Veres, G. Bencze, C. Hajdu, D. Horvath, Á. Hunyadi, F. Sikler, T. Á. Vámi, V. Veszpremi, G. Vesztergombi, N. Beni, S. Czellar, J. Karancsi, A. Makovec, J. Molnar, Z. Szillasi, M. Bartók, P. Raics, Z. L. Trocsanyi, B. Ujvari, S. Choudhury, J. R. Komaragiri, S. Bahinipati, P. Mal, K. Mandal, A. Nayak, D. K. Sahoo, S. K. Swain, S. Bansal, S. B. Beri, V. Bhatnagar, S. Chauhan, R. Chawla, N. Dhingra, R. Gupta, A. Kaur, M. Kaur, S. Kaur, R. Kumar, P. Kumari, M. Lohan, A. Mehta, S. Sharma, J. B. Singh, G. Walia, A. Bhardwaj, B. C. Choudhary, R. B. Garg, S. Keshri, A. Kumar, Ashok Kumar, S. Malhotra, M. Naimuddin, K. Ranjan, Aashaq Shah, R. Sharma, R. Bhardwaj, R. Bhattacharya, S. Bhattacharya, U. Bhawandeep, D. Bhowmik, S. Dey, S. Dutt, S. Dutta, S. Ghosh, N. Majumdar, K. Mondal, S. Mukhopadhyay, S. Nandan, A. Purohit, P. K. Rout, A. Roy, S. Roy Chowdhury, S. Sarkar, M. Sharan, B. Singh, S. Thakur, P. K. Behera, R. Chudasama, D. Dutta, V. Jha, V. Kumar, A. K. Mohanty, P. K. Netrakanti, L. M. Pant, P. Shukla, A. Topkar, T. Aziz, S. Dugad, B. Mahakud, S. Mitra, G. B. Mohanty, N. Sur, B. Sutar, S. Banerjee, S. Bhattacharya, S. Chatterjee, P. Das, M. Guchait, Sa. Jain, S. Kumar, M. Maity, G. Majumder, K. Mazumdar, N. Sahoo, T. Sarkar, N. Wickramage, S. Chauhan, S. Dube, V. Hegde, A. Kapoor, K. Kothekar, S. Pandey, A. Rane, S. Sharma, S. Chenarani, E. Eskandari Tadavani, S. M. Etesami, M. Khakzad, M. Mohammadi Najafabadi, M. Naseri, S. Paktinat Mehdiabadi, F. Rezaei Hosseinabadi, B. Safarzadeh, M. Zeinali, M. Felcini, M. Grunewald, M. Abbrescia, C. Calabria, A. Colaleo, D. Creanza, L. Cristella, N. De Filippis, M. De Palma, A. Di Florio, F. Errico, L. Fiore, A. Gelmi, G. Iaselli, S. Lezki, G. Maggi, M. Maggi, B. Marangelli, G. Miniello, S. My, S. Nuzzo, A. Pompili, G. Pugliese, R. Radogna, A. Ranieri, G. Selvaggi, A. Sharma, L. Silvestris, R. Venditti, P. Verwilligen, G. Zito, G. Abbiendi, C. Battilana, D. Bonacorsi, L. Borgonovi, S. Braibant-Giacomelli, R. Campanini, P. Capiluppi, A. Castro, F. R. Cavallo, S. S. Chhibra, G. Codispoti, M. Cuffiani, G. M. Dallavalle, F. Fabbri, A. Fanfani, D. Fasanella, P. Giacomelli, C. Grandi, L. Guiducci, S. Marcellini, G. Masetti, A. Montanari, F. L. Navarria, F. Odorici, A. Perrotta, A. M. Rossi, T. Rovelli, G. P. Siroli, N. Tosi, S. Albergo, S. Costa, A. Di Mattia, F. Giordano, R. Potenza, A. Tricomi, C. Tuve, G. Barbagli, K. Chatterjee, V. Ciulli, C. Civinini, R. D’Alessandro, E. Focardi, G. Latino, P. Lenzi, M. Meschini, S. Paoletti, L. Russo, G. Sguazzoni, D. Strom, L. Viliani, L. Benussi, S. Bianco, F. Fabbri, D. Piccolo, F. Primavera, V. Calvelli, F. Ferro, F. Ravera, E. Robutti, S. Tosi, A. Benaglia, A. Beschi, L. Brianza, F. Brivio, V. Ciriolo, M. E. Dinardo, S. Fiorendi, S. Gennai, A. Ghezzi, P. Govoni, M. Malberti, S. Malvezzi, R. A. Manzoni, D. Menasce, L. Moroni, M. Paganoni, K. Pauwels, D. Pedrini, S. Pigazzini, S. Ragazzi, T. Tabarelli de Fatis, S. Buontempo, N. Cavallo, S. Di Guida, F. Fabozzi, F. Fienga, G. Galati, A. O. M. Iorio, W. A. Khan, L. Lista, S. Meola, P. Paolucci, C. Sciacca, F. Thyssen, E. Voevodina, P. Azzi, N. Bacchetta, L. Benato, D. Bisello, A. Boletti, R. Carlin, A. Carvalho Antunes De Oliveira, P. Checchia, P. De Castro Manzano, T. Dorigo, U. Dosselli, F. Gasparini, U. Gasparini, A. Gozzelino, S. Lacaprara, P. Lujan, M. Margoni, A. T. Meneguzzo, N. Pozzobon, P. Ronchese, R. Rossin, F. Simonetto, A. Tiko, M. Zanetti, P. Zotto, G. Zumerle, A. Braghieri, A. Magnani, P. Montagna, S. P. Ratti, V. Re, M. Ressegotti, C. Riccardi, P. Salvini, I. Vai, P. Vitulo, L. Alunni Solestizi, M. Biasini, G. M. Bilei, C. Cecchi, D. Ciangottini, L. Fanò, P. Lariccia, R. Leonardi, E. Manoni, G. Mantovani, V. Mariani, M. Menichelli, A. Rossi, A. Santocchia, D. Spiga, K. Androsov, P. Azzurri, G. Bagliesi, L. Bianchini, T. Boccali, L. Borrello, R. Castaldi, M. A. Ciocci, R. Dell’Orso, G. Fedi, L. Giannini, A. Giassi, M. T. Grippo, F. Ligabue, T. Lomtadze, E. Manca, G. Mandorli, A. Messineo, F. Palla, A. Rizzi, P. Spagnolo, R. Tenchini, G. Tonelli, A. Venturi, P. G. Verdini, L. Barone, F. Cavallari, M. Cipriani, N. Daci, D. Del Re, E. Di Marco, M. Diemoz, S. Gelli, E. Longo, B. Marzocchi, P. Meridiani, G. Organtini, F. Pandolfi, R. Paramatti, F. Preiato, S. Rahatlou, C. Rovelli, F. Santanastasio, N. Amapane, R. Arcidiacono, S. Argiro, M. Arneodo, N. Bartosik, R. Bellan, C. Biino, N. Cartiglia, R. Castello, F. Cenna, M. Costa, R. Covarelli, A. Degano, N. Demaria, B. Kiani, C. Mariotti, S. Maselli, E. Migliore, V. Monaco, E. Monteil, M. Monteno, M. M. Obertino, L. Pacher, N. Pastrone, M. Pelliccioni, G. L. Pinna Angioni, A. Romero, M. Ruspa, R. Sacchi, K. Shchelina, V. Sola, A. Solano, A. Staiano, S. Belforte, M. Casarsa, F. Cossutti, G. Della Ricca, A. Zanetti, D. H. Kim, G. N. Kim, M. S. Kim, J. Lee, S. Lee, S. W. Lee, C. S. Moon, Y. D. Oh, S. Sekmen, D. C. Son, Y. C. Yang, H. Kim, D. H. Moon, G. Oh, J. A. Brochero Cifuentes, J. Goh, T. J. Kim, S. Cho, S. Choi, Y. Go, D. Gyun, S. Ha, B. Hong, Y. Jo, Y. Kim, K. Lee, K. S. Lee, S. Lee, J. Lim, S. K. Park, Y. Roh, J. Almond, J. Kim, J. S. Kim, H. Lee, K. Lee, K. Nam, S. B. Oh, B. C. Radburn-Smith, S. h. Seo, U. K. Yang, H. D. Yoo, G. B. Yu, H. Kim, J. H. Kim, J. S. H. Lee, I. C. Park, Y. Choi, C. Hwang, J. Lee, I. Yu, V. Dudenas, A. Juodagalvis, J. Vaitkus, I. Ahmed, Z. A. Ibrahim, M. A. B. Md Ali, F. Mohamad Idris, W. A. T. Wan Abdullah, M. N. Yusli, Z. Zolkapli, H. Castilla-Valdez, E. De La Cruz-Burelo, M. C. Duran-Osuna, I. Heredia-De La Cruz, R. Lopez-Fernandez, J. Mejia Guisao, R. I. Rabadan-Trejo, G. Ramirez-Sanchez, R. Reyes-Almanza, A. Sanchez-Hernandez, S. Carrillo Moreno, C. Oropeza Barrera, F. Vazquez Valencia, J. Eysermans, I. Pedraza, H. A. Salazar Ibarguen, C. Uribe Estrada, A. Morelos Pineda, D. Krofcheck, S. Bheesette, P. H. Butler, A. Ahmad, M. Ahmad, Q. Hassan, H. R. Hoorani, A. Saddique, M. A. Shah, M. Shoaib, M. Waqas, H. Bialkowska, M. Bluj, B. Boimska, T. Frueboes, M. Górski, M. Kazana, K. Nawrocki, M. Szleper, P. Traczyk, P. Zalewski, K. Bunkowski, A. Byszuk, K. Doroba, A. Kalinowski, M. Konecki, J. Krolikowski, M. Misiura, M. Olszewski, A. Pyskir, M. Walczak, P. Bargassa, C. Beirão Da Cruz E Silva, A. Di Francesco, P. Faccioli, B. Galinhas, M. Gallinaro, J. Hollar, N. Leonardo, L. Lloret Iglesias, M. V. Nemallapudi, J. Seixas, G. Strong, O. Toldaiev, D. Vadruccio, J. Varela, S. Afanasiev, P. Bunin, M. Gavrilenko, I. Golutvin, I. Gorbunov, A. Kamenev, V. Karjavin, A. Lanev, A. Malakhov, V. Matveev, P. Moisenz, V. Palichik, V. Perelygin, S. Shmatov, S. Shulha, N. Skatchkov, V. Smirnov, N. Voytishin, A. Zarubin, Y. Ivanov, V. Kim, E. Kuznetsova, P. Levchenko, V. Murzin, V. Oreshkin, I. Smirnov, D. Sosnov, V. Sulimov, L. Uvarov, S. Vavilov, A. Vorobyev, Yu. Andreev, A. Dermenev, S. Gninenko, N. Golubev, A. Karneyeu, M. Kirsanov, N. Krasnikov, A. Pashenkov, D. Tlisov, A. Toropin, V. Epshteyn, V. Gavrilov, N. Lychkovskaya, V. Popov, I. Pozdnyakov, G. Safronov, A. Spiridonov, A. Stepennov, V. Stolin, M. Toms, E. Vlasov, A. Zhokin, T. Aushev, A. Bylinkin, V. Andreev, M. Azarkin, I. Dremin, M. Kirakosyan, S. V. Rusakov, A. Terkulov, A. Baskakov, A. Belyaev, E. Boos, A. Ershov, A. Gribushin, L. Khein, V. Klyukhin, O. Kodolova, I. Lokhtin, O. Lukina, I. Miagkov, S. Obraztsov, S. Petrushanko, V. Savrin, A. Snigirev, V. Blinov, D. Shtol, Y. Skovpen, I. Azhgirey, I. Bayshev, S. Bitioukov, D. Elumakhov, A. Godizov, V. Kachanov, A. Kalinin, D. Konstantinov, P. Mandrik, V. Petrov, R. Ryutin, A. Sobol, S. Troshin, N. Tyurin, A. Uzunian, A. Volkov, A. Babaev, P. Adzic, P. Cirkovic, D. Devetak, M. Dordevic, J. Milosevic, J. Alcaraz Maestre, A. Álvarez Fernández, I. Bachiller, M. Barrio Luna, M. Cerrada, N. Colino, B. De La Cruz, A. Delgado Peris, C. Fernandez Bedoya, J. P. Fernández Ramos, J. Flix, M. C. Fouz, O. Gonzalez Lopez, S. Goy Lopez, J. M. Hernandez, M. I. Josa, D. Moran, A. Pérez-Calero Yzquierdo, J. Puerta Pelayo, I. Redondo, L. Romero, M. S. Soares, A. Triossi, C. Albajar, J. F. de Trocóniz, J. Cuevas, C. Erice, J. Fernandez Menendez, S. Folgueras, I. Gonzalez Caballero, J. R. González Fernández, E. Palencia Cortezon, S. Sanchez Cruz, P. Vischia, J. M. Vizan Garcia, I. J. Cabrillo, A. Calderon, B. Chazin Quero, J. Duarte Campderros, M. Fernandez, P. J. Fernández Manteca, A. García Alonso, J. Garcia-Ferrero, G. Gomez, A. Lopez Virto, J. Marco, C. Martinez Rivero, P. Martinez Ruiz del Arbol, F. Matorras, J. Piedra Gomez, C. Prieels, T. Rodrigo, A. Ruiz-Jimeno, L. Scodellaro, N. Trevisani, I. Vila, R. Vilar Cortabitarte, D. Abbaneo, B. Akgun, E. Auffray, P. Baillon, A. H. Ball, D. Barney, J. Bendavid, M. Bianco, A. Bocci, C. Botta, T. Camporesi, M. Cepeda, G. Cerminara, E. Chapon, Y. Chen, D. d’Enterria, A. Dabrowski, V. Daponte, A. David, M. De Gruttola, A. De Roeck, N. Deelen, M. Dobson, T. du Pree, M. Dünser, N. Dupont, A. Elliott-Peisert, P. Everaerts, F. Fallavollita, G. Franzoni, J. Fulcher, W. Funk, D. Gigi, A. Gilbert, K. Gill, F. Glege, D. Gulhan, J. Hegeman, V. Innocente, A. Jafari, P. Janot, O. Karacheban, J. Kieseler, V. Knünz, A. Kornmayer, M. Krammer, C. Lange, P. Lecoq, C. Lourenço, M. T. Lucchini, L. Malgeri, M. Mannelli, A. Martelli, F. Meijers, J. A. Merlin, S. Mersi, E. Meschi, P. Milenovic, F. Moortgat, M. Mulders, H. Neugebauer, J. Ngadiuba, S. Orfanelli, L. Orsini, F. Pantaleo, L. Pape, E. Perez, M. Peruzzi, A. Petrilli, G. Petrucciani, A. Pfeiffer, M. Pierini, F. M. Pitters, D. Rabady, A. Racz, T. Reis, G. Rolandi, M. Rovere, H. Sakulin, C. Schäfer, C. Schwick, M. Seidel, M. Selvaggi, A. Sharma, P. Silva, P. Sphicas, A. Stakia, J. Steggemann, M. Stoye, M. Tosi, D. Treille, A. Tsirou, V. Veckalns, M. Verweij, W. D. Zeuner, W. Bertl, L. Caminada, K. Deiters, W. Erdmann, R. Horisberger, Q. Ingram, H. C. Kaestli, D. Kotlinski, U. Langenegger, T. Rohe, S. A. Wiederkehr, M. Backhaus, L. Bäni, P. Berger, B. Casal, N. Chernyavskaya, G. Dissertori, M. Dittmar, M. Donegà, C. Dorfer, C. Grab, C. Heidegger, D. Hits, J. Hoss, T. Klijnsma, W. Lustermann, M. Marionneau, M. T. Meinhard, D. Meister, F. Micheli, P. Musella, F. Nessi-Tedaldi, J. Pata, F. Pauss, G. Perrin, L. Perrozzi, M. Quittnat, M. Reichmann, D. Ruini, D. A. Sanz Becerra, M. Schönenberger, L. Shchutska, V. R. Tavolaro, K. Theofilatos, M. L. Vesterbacka Olsson, R. Wallny, D. H. Zhu, T. K. Aarrestad, C. Amsler, D. Brzhechko, M. F. Canelli, A. De Cosa, R. Del Burgo, S. Donato, C. Galloni, T. Hreus, B. Kilminster, I. Neutelings, D. Pinna, G. Rauco, P. Robmann, D. Salerno, K. Schweiger, C. Seitz, Y. Takahashi, A. Zucchetta, V. Candelise, Y. H. Chang, K. y. Cheng, T. H. Doan, Sh. Jain, R. Khurana, C. M. Kuo, W. Lin, A. Pozdnyakov, S. S. Yu, P. Chang, Y. Chao, K. F. Chen, P. H. Chen, F. Fiori, W.-S. Hou, Y. Hsiung, Arun Kumar, Y. F. Liu, R.-S. Lu, E. Paganis, A. Psallidas, A. Steen, J. f. Tsai, B. Asavapibhop, K. Kovitanggoon, G. Singh, N. Srimanobhas, A. Bat, F. Boran, S. Cerci, S. Damarseckin, Z. S. Demiroglu, C. Dozen, I. Dumanoglu, S. Girgis, G. Gokbulut, Y. Guler, I. Hos, E. E. Kangal, O. Kara, U. Kiminsu, M. Oglakci, G. Onengut, K. Ozdemir, D. Sunar Cerci, B. Tali, U. G. Tok, H. Topakli, S. Turkcapar, I. S. Zorbakir, C. Zorbilmez, G. Karapinar, K. Ocalan, M. Yalvac, M. Zeyrek, I. O. Atakisi, E. Gülmez, M. Kaya, O. Kaya, S. Tekten, E. A. Yetkin, M. N. Agaras, S. Atay, A. Cakir, K. Cankocak, Y. Komurcu, B. Grynyov, L. Levchuk, F. Ball, L. Beck, J. J. Brooke, D. Burns, E. Clement, D. Cussans, O. Davignon, H. Flacher, J. Goldstein, G. P. Heath, H. F. Heath, L. Kreczko, D. M. Newbold, S. Paramesvaran, T. Sakuma, S. Seif El Nasr-storey, D. Smith, V. J. Smith, K. W. Bell, A. Belyaev, C. Brew, R. M. Brown, D. Cieri, D. J. A. Cockerill, J. A. Coughlan, K. Harder, S. Harper, J. Linacre, E. Olaiya, D. Petyt, C. H. Shepherd-Themistocleous, A. Thea, I. R. Tomalin, T. Williams, W. J. Womersley, G. Auzinger, R. Bainbridge, P. Bloch, J. Borg, S. Breeze, O. Buchmuller, A. Bundock, S. Casasso, D. Colling, L. Corpe, P. Dauncey, G. Davies, M. Della Negra, R. Di Maria, Y. Haddad, G. Hall, G. Iles, T. James, M. Komm, R. Lane, C. Laner, L. Lyons, A.-M. Magnan, S. Malik, L. Mastrolorenzo, T. Matsushita, J. Nash, A. Nikitenko, V. Palladino, M. Pesaresi, A. Richards, A. Rose, E. Scott, C. Seez, A. Shtipliyski, T. Strebler, S. Summers, A. Tapper, K. Uchida, M. Vazquez Acosta, T. Virdee, N. Wardle, D. Winterbottom, J. Wright, S. C. Zenz, J. E. Cole, P. R. Hobson, A. Khan, P. Kyberd, A. Morton, I. D. Reid, L. Teodorescu, S. Zahid, A. Borzou, K. Call, J. Dittmann, K. Hatakeyama, H. Liu, N. Pastika, C. Smith, R. Bartek, A. Dominguez, A. Buccilli, S. I. Cooper, C. Henderson, P. Rumerio, C. West, D. Arcaro, A. Avetisyan, T. Bose, D. Gastler, D. Rankin, C. Richardson, J. Rohlf, L. Sulak, D. Zou, G. Benelli, D. Cutts, M. Hadley, J. Hakala, U. Heintz, J. M. Hogan, K. H. M. Kwok, E. Laird, G. Landsberg, J. Lee, Z. Mao, M. Narain, J. Pazzini, S. Piperov, S. Sagir, R. Syarif, D. Yu, R. Band, C. Brainerd, R. Breedon, D. Burns, M. Calderon De La Barca Sanchez, M. Chertok, J. Conway, R. Conway, P. T. Cox, R. Erbacher, C. Flores, G. Funk, W. Ko, R. Lander, C. Mclean, M. Mulhearn, D. Pellett, J. Pilot, S. Shalhout, M. Shi, J. Smith, D. Stolp, D. Taylor, K. Tos, M. Tripathi, Z. Wang, F. Zhang, M. Bachtis, C. Bravo, R. Cousins, A. Dasgupta, A. Florent, J. Hauser, M. Ignatenko, N. Mccoll, S. Regnard, D. Saltzberg, C. Schnaible, V. Valuev, E. Bouvier, K. Burt, R. Clare, J. Ellison, J. W. Gary, S. M. A. Ghiasi Shirazi, G. Hanson, G. Karapostoli, E. Kennedy, F. Lacroix, O. R. Long, M. Olmedo Negrete, M. I. Paneva, W. Si, L. Wang, H. Wei, S. Wimpenny, B. R. Yates, J. G. Branson, S. Cittolin, M. Derdzinski, R. Gerosa, D. Gilbert, B. Hashemi, A. Holzner, D. Klein, G. Kole, V. Krutelyov, J. Letts, M. Masciovecchio, D. Olivito, S. Padhi, M. Pieri, M. Sani, V. Sharma, S. Simon, M. Tadel, A. Vartak, S. Wasserbaech, J. Wood, F. Würthwein, A. Yagil, G. Zevi Della Porta, N. Amin, R. Bhandari, J. Bradmiller-Feld, C. Campagnari, M. Citron, A. Dishaw, V. Dutta, M. Franco Sevilla, L. Gouskos, R. Heller, J. Incandela, A. Ovcharova, H. Qu, J. Richman, D. Stuart, I. Suarez, J. Yoo, D. Anderson, A. Bornheim, J. Bunn, J. M. Lawhorn, H. B. Newman, T. Q. Nguyen, C. Pena, M. Spiropulu, J. R. Vlimant, R. Wilkinson, S. Xie, Z. Zhang, R. Y. Zhu, M. B. Andrews, T. Ferguson, T. Mudholkar, M. Paulini, J. Russ, M. Sun, H. Vogel, I. Vorobiev, M. Weinberg, J. P. Cumalat, W. T. Ford, F. Jensen, A. Johnson, M. Krohn, S. Leontsinis, E. MacDonald, T. Mulholland, K. Stenson, K. A. Ulmer, S. R. Wagner, J. Alexander, J. Chaves, Y. Cheng, J. Chu, A. Datta, K. Mcdermott, N. Mirman, J. R. Patterson, D. Quach, A. Rinkevicius, A. Ryd, L. Skinnari, L. Soffi, S. M. Tan, Z. Tao, J. Thom, J. Tucker, P. Wittich, M. Zientek, S. Abdullin, M. Albrow, M. Alyari, G. Apollinari, A. Apresyan, A. Apyan, S. Banerjee, L. A. T. Bauerdick, A. Beretvas, J. Berryhill, P. C. Bhat, G. Bolla, K. Burkett, J. N. Butler, A. Canepa, G. B. Cerati, H. W. K. Cheung, F. Chlebana, M. Cremonesi, J. Duarte, V. D. Elvira, J. Freeman, Z. Gecse, E. Gottschalk, L. Gray, D. Green, S. Grünendahl, O. Gutsche, J. Hanlon, R. M. Harris, S. Hasegawa, J. Hirschauer, Z. Hu, B. Jayatilaka, S. Jindariani, M. Johnson, U. Joshi, B. Klima, M. J. Kortelainen, B. Kreis, S. Lammel, D. Lincoln, R. Lipton, M. Liu, T. Liu, R. Lopes De Sá, J. Lykken, K. Maeshima, N. Magini, J. M. Marraffino, D. Mason, P. McBride, P. Merkel, S. Mrenna, S. Nahn, V. O’Dell, K. Pedro, O. Prokofyev, G. Rakness, L. Ristori, A. Savoy-Navarro, B. Schneider, E. Sexton-Kennedy, A. Soha, W. J. Spalding, L. Spiegel, S. Stoynev, J. Strait, N. Strobbe, L. Taylor, S. Tkaczyk, N. V. Tran, L. Uplegger, E. W. Vaandering, C. Vernieri, M. Verzocchi, R. Vidal, M. Wang, H. A. Weber, A. Whitbeck, W. Wu, D. Acosta, P. Avery, P. Bortignon, D. Bourilkov, A. Brinkerhoff, A. Carnes, M. Carver, D. Curry, R. D. Field, I. K. Furic, S. V. Gleyzer, B. M. Joshi, J. Konigsberg, A. Korytov, K. Kotov, P. Ma, K. Matchev, H. Mei, G. Mitselmakher, K. Shi, D. Sperka, N. Terentyev, L. Thomas, J. Wang, S. Wang, J. Yelton, Y. R. Joshi, S. Linn, P. Markowitz, J. L. Rodriguez, A. Ackert, T. Adams, A. Askew, S. Hagopian, V. Hagopian, K. F. Johnson, T. Kolberg, G. Martinez, T. Perry, H. Prosper, A. Saha, A. Santra, V. Sharma, R. Yohay, M. M. Baarmand, V. Bhopatkar, S. Colafranceschi, M. Hohlmann, D. Noonan, T. Roy, F. Yumiceva, M. R. Adams, L. Apanasevich, D. Berry, R. R. Betts, R. Cavanaugh, X. Chen, S. Dittmer, O. Evdokimov, C. E. Gerber, D. A. Hangal, D. J. Hofman, K. Jung, J. Kamin, I. D. Sandoval Gonzalez, M. B. Tonjes, N. Varelas, H. Wang, Z. Wu, J. Zhang, B. Bilki, W. Clarida, K. Dilsiz, S. Durgut, R. P. Gandrajula, M. Haytmyradov, V. Khristenko, J.-P. Merlo, H. Mermerkaya, A. Mestvirishvili, A. Moeller, J. Nachtman, H. Ogul, Y. Onel, F. Ozok, A. Penzo, C. Snyder, E. Tiras, J. Wetzel, K. Yi, B. Blumenfeld, A. Cocoros, N. Eminizer, D. Fehling, L. Feng, A. V. Gritsan, W. T. Hung, P. Maksimovic, J. Roskes, U. Sarica, M. Swartz, M. Xiao, C. You, A. Al-bataineh, P. Baringer, A. Bean, S. Boren, J. Bowen, J. Castle, S. Khalil, A. Kropivnitskaya, D. Majumder, W. Mcbrayer, M. Murray, C. Rogan, C. Royon, S. Sanders, E. Schmitz, J. D. Tapia Takaki, Q. Wang, A. Ivanov, K. Kaadze, Y. Maravin, A. Modak, A. Mohammadi, L. K. Saini, N. Skhirtladze, F. Rebassoo, D. Wright, A. Baden, O. Baron, A. Belloni, S. C. Eno, Y. Feng, C. Ferraioli, N. J. Hadley, S. Jabeen, G. Y. Jeng, R. G. Kellogg, J. Kunkle, A. C. Mignerey, F. Ricci-Tam, Y. H. Shin, A. Skuja, S. C. Tonwar, D. Abercrombie, B. Allen, V. Azzolini, R. Barbieri, A. Baty, G. Bauer, R. Bi, S. Brandt, W. Busza, I. A. Cali, M. D’Alfonso, Z. Demiragli, G. Gomez Ceballos, M. Goncharov, P. Harris, D. Hsu, M. Hu, Y. Iiyama, G. M. Innocenti, M. Klute, D. Kovalskyi, Y.-J. Lee, A. Levin, P. D. Luckey, B. Maier, A. C. Marini, C. Mcginn, C. Mironov, S. Narayanan, X. Niu, C. Paus, C. Roland, G. Roland, G. S. F. Stephans, K. Sumorok, K. Tatar, D. Velicanu, J. Wang, T. W. Wang, B. Wyslouch, S. Zhaozhong, A. C. Benvenuti, R. M. Chatterjee, A. Evans, P. Hansen, S. Kalafut, Y. Kubota, Z. Lesko, J. Mans, S. Nourbakhsh, N. Ruckstuhl, R. Rusack, J. Turkewitz, M. A. Wadud, J. G. Acosta, S. Oliveros, E. Avdeeva, K. Bloom, D. R. Claes, C. Fangmeier, F. Golf, R. Gonzalez Suarez, R. Kamalieddin, I. Kravchenko, J. Monroy, J. E. Siado, G. R. Snow, B. Stieger, A. Godshalk, C. Harrington, I. Iashvili, D. Nguyen, A. Parker, S. Rappoccio, B. Roozbahani, G. Alverson, E. Barberis, C. Freer, A. Hortiangtham, A. Massironi, D. M. Morse, T. Orimoto, R. Teixeira De Lima, T. Wamorkar, B. Wang, A. Wisecarver, D. Wood, S. Bhattacharya, O. Charaf, K. A. Hahn, N. Mucia, N. Odell, M. H. Schmitt, K. Sung, M. Trovato, M. Velasco, R. Bucci, N. Dev, M. Hildreth, K. Hurtado Anampa, C. Jessop, D. J. Karmgard, N. Kellams, K. Lannon, W. Li, N. Loukas, N. Marinelli, F. Meng, C. Mueller, Y. Musienko, M. Planer, A. Reinsvold, R. Ruchti, P. Siddireddy, G. Smith, S. Taroni, M. Wayne, A. Wightman, M. Wolf, A. Woodard, J. Alimena, L. Antonelli, B. Bylsma, L. S. Durkin, S. Flowers, B. Francis, A. Hart, C. Hill, W. Ji, T. Y. Ling, W. Luo, B. L. Winer, H. W. Wulsin, S. Cooperstein, O. Driga, P. Elmer, J. Hardenbrook, P. Hebda, S. Higginbotham, A. Kalogeropoulos, D. Lange, J. Luo, D. Marlow, K. Mei, I. Ojalvo, J. Olsen, C. Palmer, P. Piroué, J. Salfeld-Nebgen, D. Stickland, C. Tully, S. Malik, S. Norberg, A. Barker, V. E. Barnes, S. Das, L. Gutay, M. Jones, A. W. Jung, A. Khatiwada, D. H. Miller, N. Neumeister, C. C. Peng, H. Qiu, J. F. Schulte, J. Sun, F. Wang, R. Xiao, W. Xie, T. Cheng, J. Dolen, N. Parashar, Z. Chen, K. M. Ecklund, S. Freed, F. J. M. Geurts, M. Guilbaud, M. Kilpatrick, W. Li, B. Michlin, B. P. Padley, J. Roberts, J. Rorie, W. Shi, Z. Tu, J. Zabel, A. Zhang, A. Bodek, P. de Barbaro, R. Demina, Y. t. Duh, T. Ferbel, M. Galanti, A. Garcia-Bellido, J. Han, O. Hindrichs, A. Khukhunaishvili, K. H. Lo, P. Tan, M. Verzetti, R. Ciesielski, K. Goulianos, C. Mesropian, A. Agapitos, J. P. Chou, Y. Gershtein, T. A. Gómez Espinosa, E. Halkiadakis, M. Heindl, E. Hughes, S. Kaplan, R. Kunnawalkam Elayavalli, S. Kyriacou, A. Lath, R. Montalvo, K. Nash, M. Osherson, H. Saka, S. Salur, S. Schnetzer, D. Sheffield, S. Somalwar, R. Stone, S. Thomas, P. Thomassen, M. Walker, A. G. Delannoy, J. Heideman, G. Riley, K. Rose, S. Spanier, K. Thapa, O. Bouhali, A. Castaneda Hernandez, A. Celik, M. Dalchenko, M. De Mattia, A. Delgado, S. Dildick, R. Eusebi, J. Gilmore, T. Huang, T. Kamon, R. Mueller, Y. Pakhotin, R. Patel, A. Perloff, L. Perniè, D. Rathjens, A. Safonov, A. Tatarinov, N. Akchurin, J. Damgov, F. De Guio, P. R. Dudero, J. Faulkner, E. Gurpinar, S. Kunori, K. Lamichhane, S. W. Lee, T. Mengke, S. Muthumuni, T. Peltola, S. Undleeb, I. Volobouev, Z. Wang, S. Greene, A. Gurrola, R. Janjam, W. Johns, C. Maguire, A. Melo, H. Ni, K. Padeken, J. D. Ruiz Alvarez, P. Sheldon, S. Tuo, J. Velkovska, Q. Xu, M. W. Arenton, P. Barria, B. Cox, R. Hirosky, M. Joyce, A. Ledovskoy, H. Li, C. Neu, T. Sinthuprasith, Y. Wang, E. Wolfe, F. Xia, R. Harr, P. E. Karchin, N. Poudyal, J. Sturdy, P. Thapa, S. Zaleski, M. Brodski, J. Buchanan, C. Caillol, D. Carlsmith, S. Dasu, L. Dodd, S. Duric, B. Gomber, M. Grothe, M. Herndon, A. Hervé, U. Hussain, P. Klabbers, A. Lanaro, A. Levine, K. Long, R. Loveless, V. Rekovic, T. Ruggles, A. Savin, N. Smith, W. H. Smith, N. Woods, CMS Collaboration

**Affiliations:** 1 Yerevan Physics Institute, Yerevan, Armenia; 2 Institut für Hochenergiephysik, Wien, Austria; 3 Institute for Nuclear Problems, Minsk, Belarus; 4 Universiteit Antwerpen, Antwerpen, Belgium; 5 Vrije Universiteit Brussel, Brussels, Belgium; 6 Université Libre de Bruxelles, Brussels, Belgium; 7 Ghent University, Ghent, Belgium; 8 Université Catholique de Louvain, Louvain-la-Neuve, Belgium; 9 Centro Brasileiro de Pesquisas Fisicas, Rio de Janeiro, Brazil; 10 Universidade do Estado do Rio de Janeiro, Rio de Janeiro, Brazil; 11 Universidade Estadual Paulista , Universidade Federal do ABC, São Paulo, Brazil; 12 Institute for Nuclear Research and Nuclear Energy, Bulgarian Academy of Sciences, Sofia, Bulgaria; 13 University of Sofia, Sofia, Bulgaria; 14 Beihang University, Beijing, China; 15 Institute of High Energy Physics, Beijing, China; 16 State Key Laboratory of Nuclear Physics and Technology, Peking University, Beijing, China; 17 Tsinghua University, Beijing, China; 18 Universidad de Los Andes, Bogota, Colombia; 19 University of Split, Faculty of Electrical Engineering, Mechanical Engineering and Naval Architecture, Split, Croatia; 20 Faculty of Science, University of Split, Split, Croatia; 21 Institute Rudjer Boskovic, Zagreb, Croatia; 22 University of Cyprus, Nicosia, Cyprus; 23 Charles University, Prague, Czech Republic; 24 Universidad San Francisco de Quito, Quito, Ecuador; 25 Academy of Scientific Research and Technology of the Arab Republic of Egypt, Egyptian Network of High Energy Physics, Cairo, Egypt; 26 National Institute of Chemical Physics and Biophysics, Tallinn, Estonia; 27 Department of Physics, University of Helsinki, Helsinki, Finland; 28 Helsinki Institute of Physics, Helsinki, Finland; 29 Lappeenranta University of Technology, Lappeenranta, Finland; 30 IRFU, CEA, Université Paris-Saclay, Gif-sur-Yvette, France; 31 Laboratoire Leprince-Ringuet, Ecole polytechnique, CNRS/IN2P3, Université Paris-Saclay, Palaiseau, France; 32 Université de Strasbourg, CNRS, IPHC UMR 7178, Strasbourg, France; 33 Centre de Calcul de l’Institut National de Physique Nucleaire et de Physique des Particules, CNRS/IN2P3, Villeurbanne, France; 34 Université de Lyon, Université Claude Bernard Lyon 1, CNRS-IN2P3, Institut de Physique Nucléaire de Lyon, Villeurbanne, France; 35 Georgian Technical University, Tbilisi, Georgia; 36 Tbilisi State University, Tbilisi, Georgia; 37 RWTH Aachen University, I. Physikalisches Institut, Aachen, Germany; 38 RWTH Aachen University, III. Physikalisches Institut A, Aachen, Germany; 39 RWTH Aachen University, III. Physikalisches Institut B, Aachen, Germany; 40 Deutsches Elektronen-Synchrotron, Hamburg, Germany; 41 University of Hamburg, Hamburg, Germany; 42 Karlsruher Institut fuer Technologie, Karlsruhe, Germany; 43 Institute of Nuclear and Particle Physics (INPP), NCSR Demokritos, Aghia Paraskevi, Greece; 44 National and Kapodistrian University of Athens, Athens, Greece; 45 National Technical University of Athens, Athens, Greece; 46 University of Ioánnina, Ioannina, Greece; 47 MTA-ELTE Lendület CMS Particle and Nuclear Physics Group, Eötvös Loránd University, Budapest, Hungary; 48 Wigner Research Centre for Physics, Budapest, Hungary; 49 Institute of Nuclear Research ATOMKI, Debrecen, Hungary; 50 Institute of Physics, University of Debrecen, Debrecen, Hungary; 51 Indian Institute of Science (IISc), Bangalore, India; 52 National Institute of Science Education and Research, HBNI, Bhubaneswar, India; 53 Panjab University, Chandigarh, India; 54 University of Delhi, Delhi, India; 55 Saha Institute of Nuclear Physics, HBNI, Kolkata, India; 56 Indian Institute of Technology Madras, Madras, India; 57 Bhabha Atomic Research Centre, Mumbai, India; 58 Tata Institute of Fundamental Research-A, Mumbai, India; 59 Tata Institute of Fundamental Research-B, Mumbai, India; 60 Indian Institute of Science Education and Research (IISER), Pune, India; 61 Institute for Research in Fundamental Sciences (IPM), Tehran, Iran; 62 University College Dublin, Dublin, Ireland; 63 INFN Sezione di Bari , Università di Bari , Politecnico di Bari, Bari, Italy; 64 INFN Sezione di Bologna , Università di Bologna, Bologna, Italy; 65 INFN Sezione di Catania , Università di Catania, Catania, Italy; 66 INFN Sezione di Firenze , Università di Firenze, Firenze, Italy; 67 INFN Laboratori Nazionali di Frascati, Frascati, Italy; 68 INFN Sezione di Genova , Università di Genova, Genoa, Italy; 69 INFN Sezione di Milano-Bicocca , Università di Milano-Bicocca, Milan, Italy; 70 INFN Sezione di Napoli , Università di Napoli ’Federico II’ , Napoli, Italy, Università della Basilicata , Potenza, Italy, Università G. Marconi, Rome, Italy; 71 INFN Sezione di Padova , Università di Padova , Padova, Italy, Università di Trento, Trento, Italy; 72 INFN Sezione di Pavia , Università di Pavia, Pavia, Italy; 73 INFN Sezione di Perugia , Università di Perugia, Perugia, Italy; 74 INFN Sezione di Pisa , Università di Pisa , Scuola Normale Superiore di Pisa, Pisa, Italy; 75 INFN Sezione di Roma , Sapienza Università di Roma, Rome, Italy; 76 INFN Sezione di Torino , Università di Torino , Torino, Italy, Università del Piemonte Orientale, Novara, Italy; 77 INFN Sezione di Trieste , Università di Trieste, Trieste, Italy; 78 Kyungpook National University, Daegu, Korea; 79 Chonnam National University, Institute for Universe and Elementary Particles, Kwangju, Korea; 80 Hanyang University, Seoul, Korea; 81 Korea University, Seoul, Korea; 82 Seoul National University, Seoul, Korea; 83 University of Seoul, Seoul, Korea; 84 Sungkyunkwan University, Suwon, Korea; 85 Vilnius University, Vilnius, Lithuania; 86 National Centre for Particle Physics, Universiti Malaya, Kuala Lumpur, Malaysia; 87 Centro de Investigacion y de Estudios Avanzados del IPN, Mexico City, Mexico; 88 Universidad Iberoamericana, Mexico City, Mexico; 89 Benemerita Universidad Autonoma de Puebla, Puebla, Mexico; 90 Universidad Autónoma de San Luis Potosí, San Luis Potosí, Mexico; 91 University of Auckland, Auckland, New Zealand; 92 University of Canterbury, Christchurch, New Zealand; 93 National Centre for Physics, Quaid-I-Azam University, Islamabad, Pakistan; 94 National Centre for Nuclear Research, Swierk, Poland; 95 Institute of Experimental Physics, Faculty of Physics, University of Warsaw, Warsaw, Poland; 96 Laboratório de Instrumentação e Física Experimental de Partículas, Lisbon, Portugal; 97 Joint Institute for Nuclear Research, Dubna, Russia; 98 Petersburg Nuclear Physics Institute, Gatchina (St. Petersburg), Russia; 99 Institute for Nuclear Research, Moscow, Russia; 100 Institute for Theoretical and Experimental Physics, Moscow, Russia; 101 Moscow Institute of Physics and Technology, Moscow, Russia; 102 P.N. Lebedev Physical Institute, Moscow, Russia; 103 Skobeltsyn Institute of Nuclear Physics, Lomonosov Moscow State University, Moscow, Russia; 104 Novosibirsk State University (NSU), Novosibirsk, Russia; 105 Institute for High Energy Physics of National Research Centre ‘Kurchatov Institute’, Protvino, Russia; 106 National Research Tomsk Polytechnic University, Tomsk, Russia; 107 Faculty of Physics and Vinca Institute of Nuclear Sciences, University of Belgrade, Belgrade, Serbia; 108 Centro de Investigaciones Energéticas Medioambientales y Tecnológicas (CIEMAT), Madrid, Spain; 109 Universidad Autónoma de Madrid, Madrid, Spain; 110 Universidad de Oviedo, Oviedo, Spain; 111 Instituto de Física de Cantabria (IFCA), CSIC-Universidad de Cantabria, Santander, Spain; 112 CERN, European Organization for Nuclear Research, Geneva, Switzerland; 113 Paul Scherrer Institut, Villigen, Switzerland; 114 ETH Zurich-Institute for Particle Physics and Astrophysics (IPA), Zurich, Switzerland; 115 Universität Zürich, Zurich, Switzerland; 116 National Central University, Chung-Li, Taiwan; 117 National Taiwan University (NTU), Taipei, Taiwan; 118 Chulalongkorn University, Faculty of Science, Department of Physics, Bangkok, Thailand; 119 Çukurova University, Physics Department, Science and Art Faculty, Adana, Turkey; 120 Middle East Technical University, Physics Department, Ankara, Turkey; 121 Bogazici University, Istanbul, Turkey; 122 Istanbul Technical University, Istanbul, Turkey; 123 Institute for Scintillation Materials of National Academy of Science of Ukraine, Kharkov, Ukraine; 124 National Scientific Center, Kharkov Institute of Physics and Technology, Kharkov, Ukraine; 125 University of Bristol, Bristol, UK; 126 Rutherford Appleton Laboratory, Didcot, UK; 127 Imperial College, London, UK; 128 Brunel University, Uxbridge, UK; 129 Baylor University, Waco, USA; 130 Catholic University of America, Washington, DC USA; 131 The University of Alabama, Tuscaloosa, USA; 132 Boston University, Boston, USA; 133 Brown University, Providence, USA; 134 University of California, Davis, Davis, USA; 135 University of California, Los Angeles, USA; 136 University of California, Riverside, Riverside, USA; 137 University of California, San Diego, La Jolla, USA; 138 Department of Physics, University of California, Santa Barbara, Santa Barbara, USA; 139 California Institute of Technology, Pasadena, USA; 140 Carnegie Mellon University, Pittsburgh, USA; 141 University of Colorado Boulder, Boulder, USA; 142 Cornell University, Ithaca, USA; 143 Fermi National Accelerator Laboratory, Batavia, USA; 144 University of Florida, Gainesville, USA; 145 Florida International University, Miami, USA; 146 Florida State University, Tallahassee, USA; 147 Florida Institute of Technology, Melbourne, USA; 148 University of Illinois at Chicago (UIC), Chicago, USA; 149 The University of Iowa, Iowa City, USA; 150 Johns Hopkins University, Baltimore, USA; 151 The University of Kansas, Lawrence, USA; 152 Kansas State University, Manhattan, USA; 153 Lawrence Livermore National Laboratory, Livermore, USA; 154 University of Maryland, College Park, USA; 155 Massachusetts Institute of Technology, Cambridge, USA; 156 University of Minnesota, Minneapolis, USA; 157 University of Mississippi, Oxford, USA; 158 University of Nebraska-Lincoln, Lincoln, USA; 159 State University of New York at Buffalo, Buffalo, USA; 160 Northeastern University, Boston, USA; 161 Northwestern University, Evanston, USA; 162 University of Notre Dame, Notre Dame, USA; 163 The Ohio State University, Columbus, USA; 164 Princeton University, Princeton, USA; 165 University of Puerto Rico, Mayaguez, USA; 166 Purdue University, West Lafayette, USA; 167 Purdue University Northwest, Hammond, USA; 168 Rice University, Houston, USA; 169 University of Rochester, Rochester, USA; 170 The Rockefeller University, New York, USA; 171 Rutgers, The State University of New Jersey, Piscataway, USA; 172 University of Tennessee, Knoxville, USA; 173 Texas A&M University, College Station, USA; 174 Texas Tech University, Lubbock, USA; 175 Vanderbilt University, Nashville, USA; 176 University of Virginia, Charlottesville, USA; 177 Wayne State University, Detroit, USA; 178 University of Wisconsin-Madison, Madison, WI USA; 179 CERN, 1211 Geneva 23, Switzerland

**Keywords:** CMS, UPC, photoproduction, Y, pPb

## Abstract

The exclusive photoproduction of $\mathrm {\Upsilon }\mathrm {(nS)}$ meson states from protons, $\gamma \mathrm {p} \rightarrow \mathrm {\Upsilon }\mathrm {(nS)} \,\mathrm {p}$ (with $\mathrm {n}=1,2,3$), is studied in ultraperipheral $\mathrm {p}$Pb collisions at a centre-of-mass energy per nucleon pair of $\sqrt{\smash [b]{s_{_{\mathrm {NN}}}}} = 5.02\,\text {TeV}$. The measurement is performed using the $\mathrm {\Upsilon }\mathrm {(nS)} \rightarrow \mu ^+\mu ^-$ decay mode, with data collected by the CMS experiment corresponding to an integrated luminosity of 32.6$\,\text {nb}^{-1}$. Differential cross sections as functions of the $\mathrm {\Upsilon }\mathrm {(nS)}$ transverse momentum squared $p_{\mathrm {T}} ^2$, and rapidity *y*, are presented. The $\mathrm {\Upsilon (1S)}$ photoproduction cross section is extracted in the rapidity range $|y |< 2.2$, which corresponds to photon–proton centre-of-mass energies in the range $91<W_{\gamma \mathrm {p}} <826\,\text {GeV}$. The data are compared to theoretical predictions based on perturbative quantum chromodynamics and to previous measurements.

## Introduction

**Fig. 1:**
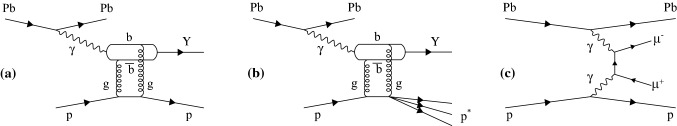
Diagrams representing **a** exclusive $\mathrm {\Upsilon }$ photoproduction, **b** proton dissociative , or “semiexclusive”, $\mathrm {\Upsilon }$ photoproduction, and **c** exclusive dimuon QED continuum production in $\mathrm {p}$Pb collisions

This paper reports a first measurement of the exclusive photoproduction of $\mathrm {\Upsilon }$ mesons from protons in $\mathrm {p}$Pb collisions at a nucleon–nucleon centre-of-mass energy of $\sqrt{\smash [b]{s_{_{\mathrm {NN}}}}} = 5.02\,\text {TeV}$, performed at the CERN LHC with the CMS detector. Exclusive photoproduction of vector mesons can be studied at the LHC in ultraperipheral collisions (UPCs) of protons and/or ions occurring at impact parameters larger than the sum of their radii, thereby largely suppressing their hadronic interaction [1]. In such UPCs, one of the incoming hadrons emits a quasi-real photon that converts into a $\mathrm {q} \overline{\mathrm {q}}$ (vector meson) bound state following a colour-singlet gluon exchange with the other “target” proton or ion [2, 3]. Since the incoming hadrons remain intact after the interaction and only the vector meson is produced in the event, the process is called “exclusive”. Given that the photon flux scales with the square of the emitting electric charge, the radiation of quasi-real photons from the Pb ion is strongly enhanced compared to that from the proton. Figure 1a shows the dominant diagram for the exclusive $\mathrm {\Upsilon }$ photoproduction signal in $\mathrm {p}$Pb collisions, $\mathrm {p}\mathrm {Pb} \rightarrow (\gamma \mathrm {p})\mathrm {Pb}\rightarrow \mathrm {p}\,\mathrm {\Upsilon }\,\mathrm {Pb}$. If the $\mathrm {\Upsilon }$ photoproduction is followed by the proton breakup, the process is called “semiexclusive” (Fig. 1b). The exchanged photon can also interact with a photon radiated from the proton [1, 4]. This two-photon collision can produce an exclusive dimuon state, as shown in Fig. 1c. Since we are interested in studying exclusive $\mathrm {\Upsilon }$ production via its dimuon decay, the latter quantum electrodynamics (QED) continuum production constitutes a background process.

The study of exclusive photoproduction of quarkonia offers a clean probe of the target hadron structure [1, 3, 5], with the large mass of the $\mathrm {J}/\psi$ and $\mathrm {\Upsilon }$ mesons providing a hard scale for calculations based on perturbative quantum chromodynamics (pQCD) [6–9]. In the kinematic region studied here, the photoproduction of $\mathrm {J}/\psi$ and $\mathrm {\Upsilon }$ mesons from protons is sensitive to generalized parton distributions (GPDs), which can be approximated by the square of the gluon density in the proton [6–19]. Experimentally, exclusive $\mathrm {J}/\psi$ and $\mathrm {\Upsilon }$ photoproduction cross sections have been observed to rise with photon–proton centre-of-mass energy $W_{\gamma \mathrm {p}}$, following a power-law dependence $W_{\gamma \mathrm {p}} ^{\delta }$ with $\delta =0.7$–1.2 [20, 21]. This reflects the steep rise of the underlying gluon density in the proton for decreasing values of the momentum fraction *x* of the proton carried by the struck parton. The dependence of the exclusive vector meson photoproduction cross section on the squared four-momentum transfer at the proton vertex *t*, parameterized at low values of $|t |$ with an exponential function of the form $\exp (-b|t |)$ [20, 22–24], has also often been studied; the *b* slope parameter provides valuable information on the parton transverse density profile of the proton [7, 8, 25].

Exclusive $\mathrm {\Upsilon }$ meson photoproduction was first observed in electron-proton collisions at HERA [20–22, 24] with the quasi-real photon radiated from the electron. At the CERN LHC, the LHCb [26–28], CMS [29], and ALICE [30–33] experiments have measured exclusive photoproduction of $\mathrm {J}/\psi$ mesons in ultraperipheral proton-proton and nuclear collisions. The LHCb experiment has also reported the measurement of the exclusive $\mathrm {\Upsilon }$ photoproduction cross section in $\mathrm {p}\mathrm {p}$ collisions at $\sqrt{s} = 7$ and 8$\,\text {TeV}$  [34]. The larger mass of the $\mathrm {\Upsilon }$ meson provides a larger perturbative scale at which the gluon distribution in the proton is sampled, and thereby reduces theoretical uncertainties in pQCD calculations. This allows the data to constrain the gluon distributions at low values of Bjorken *x* in global PDF fits for the first time [35]. The present paper reports the measurement of $\mathrm {\Upsilon }$ photoproduction in $\mathrm {p}$Pb UPCs that probes the gluon density of the proton in the region $x= m_{\mathrm {\Upsilon }}^2/W_{\gamma \mathrm {p}} ^2= 10^{-4}$–$10^{-2}$ [3], where $m_{\mathrm {\Upsilon }}$ is the $\mathrm {\Upsilon }$ meson mass. This CMS measurement spans a previously unexplored low-*x* region between the HERA and LHCb data, and provides additional experimental insights on the gluon content in the proton. In this low-*x* regime, nonlinear QCD effects (gluon recombination) may become important, possibly leading to the saturation of the parton distribution functions (PDFs) [36–38].

The measurements presented here are carried out using the $\mu ^{+}\mu ^{-}$ decays of the $\mathrm {\Upsilon }\mathrm {(nS)}$ ($\mathrm {n}=1$, 2, 3) bottomonium mesons in the rapidity range $|y |<2.2$ in the laboratory frame. These include differential cross sections as functions of the $\mathrm {\Upsilon }$ rapidity and transverse momentum squared $p_{\mathrm {T}} ^2$ (which approximates the absolute value of the four-momentum transfer squared at the proton vertex, $|t |$), as well as the total $\mathrm {\Upsilon (1S)}$ cross section as a function of $W_{\gamma \mathrm {p}}$. The results are compared to previous measurements and to theoretical predictions based on leading order (LO) and next-to-leading-order (NLO) pQCD calculations [10], as well as on colour dipole [15, 16] and gluon saturation [15–19] approaches.

## Experimental setup

The central feature of the CMS apparatus is a superconducting solenoid of 6$\text { m}$ internal diameter, providing a magnetic field of 3.8$\text { T}$. Within the solenoid volume are a silicon pixel and strip tracker, a lead tungstate crystal electromagnetic calorimeter (ECAL), and a brass and scintillator hadron calorimeter (HCAL), each composed of a barrel and two endcap sections. The silicon pixel and strip tracker measures charged-particle trajectories within the pseudorapidity range $|\eta |< 2.5$. It consists of 66 million pixel and 10 million strip sensor elements. For charged particles with $1< p_{\mathrm {T}} < 10\,\text {GeV}$ and $|\eta | < 1.4$, the track resolutions are typically 1.5% in $p_{\mathrm {T}}$  [39].

Muons are measured in gas-ionisation detectors embedded in the steel flux-return yoke outside the solenoid over the range $|\eta |< 2.4$, with detection planes based on three technologies: drift tubes, cathode strip chambers, and resistive-plate chambers. The reconstruction algorithm considers all tracks in the silicon tracker and identifies them as muons by looking for compatible signatures in the calorimeters and in the muon system. Because of the strong magnetic field and the fine granularity of the tracker, the muon $p_{\mathrm {T}}$ measurement based on information from the tracker alone has a good resolution [40].

Extensive forward calorimetry, based on Cherenkov radiation detectors, complements the coverage provided by the barrel and endcap calorimeters. Two hadron forward (HF) calorimeters, consisting of iron absorbers and embedded radiation-hard quartz fibres, cover $2.9< |\eta |< 5.2$, and two zero-degree calorimeters (ZDCs), with alternating layers of tungsten and quartz fibers, are sensitive to neutrons and photons with $|\eta |> 8.3$ [41].

The data are collected with a two-level trigger system. The first level of the CMS trigger system, composed of custom hardware processors, uses information from the calorimeters and muon detectors to select the most interesting events [42]. The high-level trigger (HLT) processor farm runs a version of the full event reconstruction software optimized for fast processing. A more detailed description of the CMS detector, together with a definition of the coordinate system used and the relevant kinematic variables, can be found in Ref. [43].

## Data sample and Monte Carlo simulation

The data set used in this analysis corresponds to 32.6$\,\text {nb}^{-1}$ of integrated luminosity collected in $\mathrm {p}$Pb collisions by the CMS experiment in 2013, with beam energies of 4$\,\text {TeV}$ for the protons and 1.58$\,\text {TeV}$ per nucleon for the lead nuclei, resulting in a nucleon–nucleon centre-of-mass energy of $\sqrt{\smash [b]{s_{_{\mathrm {NN}}}}} = 5.02\,\text {TeV}$. The data are the sum of the collected $\mathrm {p}$Pb and Pb$\mathrm {p}$ collision samples, with the incoming Pb ion going in the $+z$ and $-z$ beam directions, corresponding to integrated luminosities of 18.8 and 13.8$\,\text {nb}^{-1}$, respectively.

The photon–proton centre-of-mass energy, $W_{\gamma \mathrm {p}}$, is related to the rapidity *y* of the $\mathrm {\Upsilon }$ meson in the laboratory frame by $W_{\gamma \mathrm {p}} ^{2}=2E_\mathrm {p}m_{\mathrm {\Upsilon }} \exp (\pm y)$, where $E_\mathrm {p}$ is the proton energy, and the $+(-)$ sign corresponds to the $\mathrm {p}$Pb (Pb$\mathrm {p}$) beam configuration. This formula, derived neglecting the transverse momenta involved in the interaction, approximates the true value of $W_{\gamma \mathrm {p}}$ to better than 1 per mille in the $W_{\gamma \mathrm {p}}$ range of this measurement. The data span the range $91< W_{\gamma \mathrm {p}} < 826\,\text {GeV}$, with the limits given by the maximum and minimum rapidities, over $|y |<2.2$, of the $\mathrm {\Upsilon }$ mesons. Because the CMS detector is symmetric along *z*, the $\mathrm {p}$Pb and Pb$\mathrm {p}$ data samples are merged in this analysis after changing the sign of $p_{z}$ of the final state particles in the Pb$\mathrm {p}$ sample.

The starlight (v3.07) [44, 45] Monte Carlo (MC) event generator is used to simulate exclusive $\mathrm {\Upsilon }\mathrm {(nS)}$ photoproduction events (Fig. 1a) and the exclusive QED background (Fig. 1c). The starlight MC assumes that the photon flux from the incoming hadron(s) is described by the Weizsäcker–Williams equivalent photon approximation [46, 47], and uses an empirical fit of the exclusive vector meson photoproduction cross sections to the existing HERA $\gamma \mathrm {p}$ data. In the $\mathrm {\Upsilon }\mathrm {(nS)}$ sample, two contributions are simulated, with the photon being emitted either from the Pb ion or from the proton. The $\gamma \mathrm {p}$ events where the photon is emitted from the Pb ion constitute the signal, while the small fraction of $\gamma \mathrm {Pb}$ events with the photon emitted from the proton is treated as a background. The signal events in the starlight MC are simulated assuming a $|t |$-differential cross section following an $\exp (-b|t |)$ dependence, and a power law dependence of the cross section on the photon–proton centre-of-mass energy, $W_{\gamma \mathrm {p}} ^{\delta }$, with the exponent $\delta$. In this study, the *b* and $\delta$ parameters are tuned to reproduce the data through a reweighting procedure described in Sect. 4. The backgrounds from inclusive and semiexclusive $\mathrm {\Upsilon }$ and dimuon production processes are obtained using templates derived from control samples in the data, as explained in the next section. All simulated events are passed through the Geant4-based [48–50] detector simulation and the event reconstruction chain of CMS.

## Event selection and background estimation

The $\mathrm {\Upsilon }\mathrm {(nS)}$ states are studied in their dimuon decay channel. The UPC dimuon events are selected at the trigger level with a dedicated HLT algorithm, requiring at least one muon and at least one, but not more than six, tracks in the event. At the offline level, additional selection criteria for muon quality requirements, are applied [40, 51]. In order to minimize the uncertainties related to the low-$p_{\mathrm {T}}$ muon reconstruction inefficiencies, muons with $p_{\mathrm {T}} ^{\mu }>3.3\,\text {GeV}$ are selected in the region $|\eta ^{\mu } |<2.2$ in the laboratory frame. Exclusive events are selected by requiring two opposite-charge muons with a single vertex and no extra charged particles with $p_{\mathrm {T}} >0.1\,\text {GeV}$ associated with it. In addition, no energy deposits in the HF calorimeters are allowed. This is achieved by requiring that the largest HF tower energy deposit be smaller than 5$\,\text {GeV}$. The HF energy threshold is set to be larger than the detector noise, and is determined from the energy distributions collected in dedicated data taking with no LHC beams. Furthermore, the rapidity of the muon pair is required to be in the region $|y |<2.2$ in the laboratory frame. Only events with the $p_{\mathrm {T}}$ of the muon pair between 0.1 and 1$\,\text {GeV}$ are considered, thereby reducing the contamination from QED pairs at very low $p_{\mathrm {T}}$ and from $\mathrm {\Upsilon }$ meson production in inclusive and semiexclusive (where the proton dissociates into a low-mass hadronic system, Fig. 1b) processes that dominate the region of large dimuon $p_{\mathrm {T}} >1\,\text {GeV}$.

**Fig. 2:**
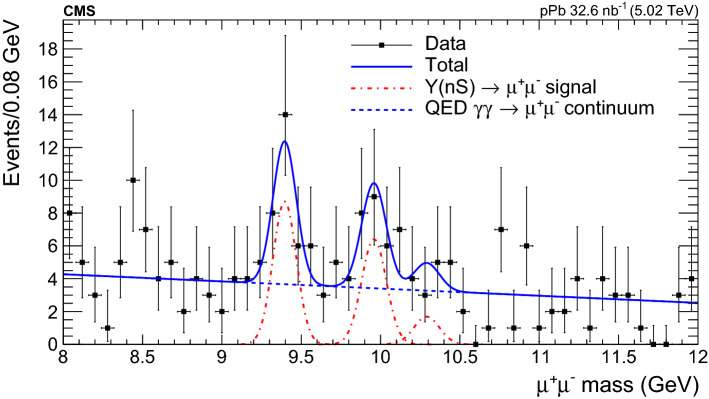
Invariant mass distribution of the exclusive muon pair candidates in the range $8< m_{\mu ^{+}\mu ^{-}}< 12\,\text {GeV}$ that pass all the selection criteria, fitted to a linear function for the two-photon QED continuum (blue dashed line) plus three Gaussian distributions corresponding to the $\mathrm {\Upsilon (1S)}$, $\mathrm {\Upsilon (2S)}$, and $\mathrm {\Upsilon (3S)}$ mesons (dashed-dotted-red curves)

Figure 2 shows the invariant mass distribution of $\mu ^{+}\mu ^{-}$ pairs in the range between 8 and 12$\,\text {GeV}$ that satisfy the selection criteria described above. An unbinned likelihood fit to the spectrum is performed using RooFit [52] with a linear function to describe the QED $\gamma \gamma \rightarrow \mu ^+\mu ^-$ continuum background, where the background slope parameter is fixed to the starlight$\gamma \gamma \rightarrow \mu ^+\mu ^-$ simulation, plus three Gaussian functions for the three $\mathrm {\Upsilon }$ signal peaks, since the natural widths of the $\mathrm {\Upsilon }\mathrm {(nS)}$ states are much smaller than their (Gaussian) experimental invariant mass resolutions. The six free parameters of the fit are the normalizations of the background and the three signal peaks, as well as the mass and the width of the $\mathrm {\Upsilon (1S)}$ resonance. The $\mathrm {\Upsilon (2S)}-\mathrm {\Upsilon (1S)}$ and $\mathrm {\Upsilon (3S)}-\mathrm {\Upsilon (1S)}$ mass differences are fixed to their PDG values [53], while the widths of $\mathrm {\Upsilon (2S)}$ and $\mathrm {\Upsilon (3S)}$ are expressed in terms of the $\mathrm {\Upsilon (1S)}$ width scaled by the ratio of their masses. The parameters describing the background plus the $\mathrm {\Upsilon (1S)}$ and $\mathrm {\Upsilon (2S)}$ resonances do not change if the $\mathrm {\Upsilon (3S)}$ signal is neglected in the fit. The statistical significance of the $\mathrm {\Upsilon (1S)}+\mathrm {\Upsilon (2S)}$ peaks over the background is $3.9 \sigma$. The apparent excess at $8.5\,\text {GeV}$ has a local significance of $1.6\sigma$, and is consistent with a statistical fluctuation. Because of the overall small number of events in the data sample, a determination of the separate $\mathrm {\Upsilon }\mathrm {(nS)}$ differential cross sections by fitting the invariant mass spectrum in each $p_{\mathrm {T}} ^2$ and *y* bin leads to results with large statistical fluctuations. Instead, the cross sections are extracted by adding up the events, after background subtraction, in the 9.1–10.6$\,\text {GeV}$ mass region corresponding to the three $\mathrm {\Upsilon }$ states combined, and the $\mathrm {\Upsilon (1S)}$ yield is derived from the $\mathrm {\Upsilon (1S)}$/$\mathrm {\Upsilon }\mathrm {(sum)}$ ratio, where $\mathrm {\Upsilon }\mathrm {(sum)} =\mathrm {\Upsilon (1S)}+\mathrm {\Upsilon (2S)}+\mathrm {\Upsilon (3S)}$, as described in Sect. 5.

**Fig. 3:**
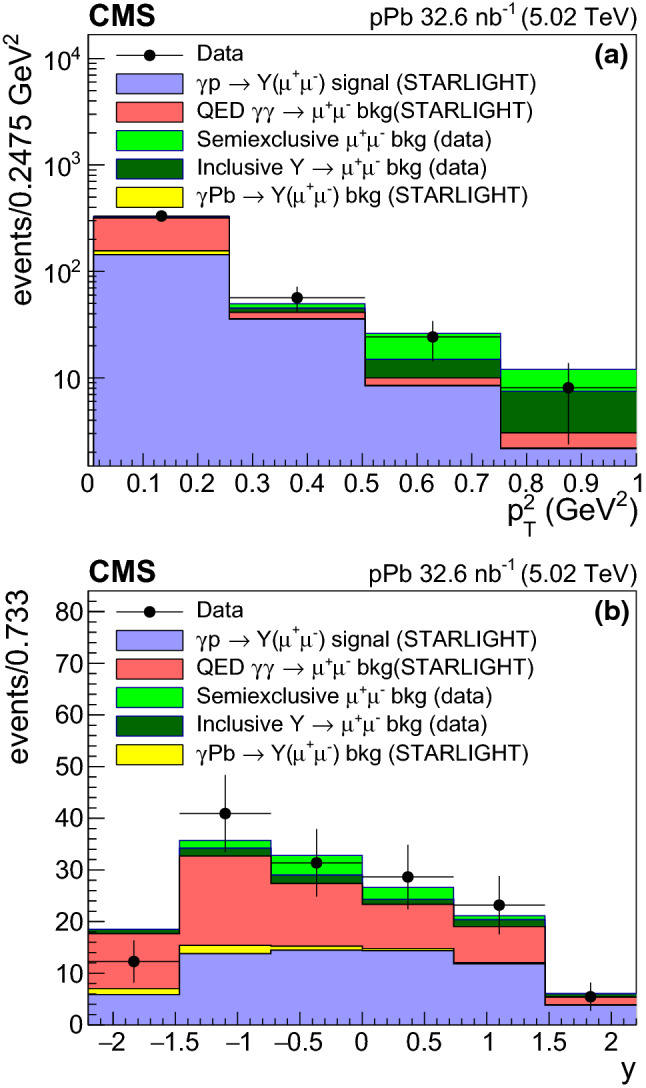
Distributions of the **a** transverse momentum squared $p_{\mathrm {T}} ^2$, and **b** rapidity *y* of exclusive muon pairs with invariant mass $9.1<m_{\mu ^{+}\mu ^{-}}<10.6~\,\text {GeV}$ after all selection criteria have been applied. Both distributions are compared to the expectations of signal and background contributions discussed in the text

Figure 3 shows the dimuon $p_{\mathrm {T}} ^2$ and rapidity distributions in the invariant mass interval $9.1<m_{\mu ^{+}\mu ^{-}}<10.6\,\text {GeV}$ for events passing all the selection criteria for the combined $\mathrm {p}$Pb and Pb$\mathrm {p}$ samples. The data, uncorrected for detector effects, are compared to the starlight simulation for exclusive $\mathrm {\Upsilon }\mathrm {(nS)}$ and QED dimuon production, normalized to the recorded integrated luminosity, together with the inclusive and semiexclusive backgrounds derived from the data themselves as discussed below. The simulated $\mathrm {\Upsilon }\mathrm {(nS)}$ events from starlight are shown separately for the $\gamma \mathrm {p}$ and $\gamma \mathrm {Pb}$ processes; the latter (with much smaller cross sections) are considered as a background in this analysis. The $\mathrm {\Upsilon }\mathrm {(nS)}$ events generated with starlight are reweighted to describe the data, using the parameters $b = 5.8\,\text {GeV} ^{-2}$ for the $|t |$ distribution slope, and $\delta =0.99$ for the cross section energy dependence. These parameters minimize the $\chi ^2$ goodness-of-fit value calculated using the data and MC distributions of Fig. 3. The minimization is performed as a function of the rapidity simultaneously for the $\gamma \mathrm {p}$ and $\gamma \mathrm {Pb}$ samples, and as a function of $p_{\mathrm {T}} ^2$ for the $\gamma \mathrm {p}$ events. For $\gamma \mathrm {Pb}$ events, the default starlight$p_{\mathrm {T}}$ spectrum is used.

In order to extract the exclusive $\gamma \mathrm {p} \rightarrow \mathrm {\Upsilon }(\mu ^+\mu ^-) \mathrm {p}$ signal events, the exclusive QED and other nonexclusive background contributions need to be subtracted. The QED $\gamma \gamma \rightarrow \mu ^+\mu ^-$ continuum under the $\mathrm {\Upsilon }\mathrm {(nS)}$ peaks is estimated with the starlight MC simulation. The absolute prediction of the cross section from this generator is cross-checked by comparing the data and the simulation in a control region, corresponding to small values of dimuon $p_{\mathrm {T}}$, $p_{\mathrm {T}} <0.15\,\text {GeV}$, and away from the $\mathrm {\Upsilon }$ resonances, $8<m_{\mu ^{+}\mu ^{-}}<9.1\,\text {GeV}$ and $10.6<m_{\mu ^{+}\mu ^{-}}<12\,\text {GeV}$, where the QED process is dominant. The ratio of the measured yields in the data to those from the starlight MC in the control region is measured to be $1.03 \pm 0.10$, confirming that this event generator reproduces the QED background well, as observed previously in $\mathrm {p}$Pb and PbPb collisions at the LHC [29–32]. The QED contribution, estimated from the starlight MC in the signal region, amounts to 40% (64 and 8% in the lowest and highest dimuon $p_{\mathrm {T}} ^2$ bins of the corresponding differential cross section, respectively).

Backgrounds to the exclusive $\mathrm {\Upsilon }\rightarrow \mu ^+\mu ^-$ signal also originate from semiexclusive and inclusive $\mathrm {\Upsilon }$ meson and Drell–Yan (DY) continuum production, where any additional hadronic activity falls outside the detector acceptance or below the detection thresholds. These background contributions are estimated from the data, by removing selectively the neutral or charged exclusivity requirements. A template dominated by semiexclusive contributions is constructed using events with only two muon tracks in the tracker accompanied by at least one HF tower having an energy deposit larger than the noise threshold of 5$\,\text {GeV}$, in the direction of the outgoing proton. Events with two muons satisfying the selection criteria, but with at least one additional track with $p_{\mathrm {T}} >0.1\,\text {GeV}$, are used to build a template dominated by inclusive DY production events. The normalizations of the two templates are obtained from a fit to the measured $p_{\mathrm {T}} ^2$ distribution extended up to $p_{\mathrm {T}} ^2=10\,\text {GeV} ^2$, where proton dissociation and inclusive events dominate, as seen in the tail of the distribution of Fig. 3a. The combination of the normalized inclusive and semiexclusive templates describes the region of high dimuon $p_{\mathrm {T}} ^2$ well in the data in all four *y* bins used for the cross section extraction. The overall fraction of both backgrounds in the signal sample is estimated to be 11% (3 and 48% in the lowest and highest dimuon $p_{\mathrm {T}} ^2$ bin, respectively). As an extra cross check of the nonexclusive background subtraction, the signal extraction is repeated by requiring in addition no neutron detection in the ZDC calorimeters [29]. The extracted yield of exclusive $\mathrm {\Upsilon }$ candidates at low $p_{\mathrm {T}}$ is found to be consistent with the nominal results without applying the ZDC veto requirement, thereby confirming the efficiency of the nonexclusive background rejection.

An additional background in this analysis originates from a small contribution of exclusive $\gamma \mathrm {Pb} \rightarrow \mathrm {\Upsilon }\mathrm {Pb}$ events. It is estimated using the reweighted starlight$\mathrm {\Upsilon }$ MC sample, and amounts to 6% (16 and 1% in the lowest and highest dimuon $p_{\mathrm {T}} ^2$ bin, respectively) of the $\gamma \mathrm {p}$ MC signal. Relative to the data, this contribution amounts to 3% (5 and 1% at the lowest and highest dimuon $p_{\mathrm {T}} ^2$ bin, respectively). These simulation-based fractions are used to subtract the $\gamma \mathrm {Pb} \rightarrow \mathrm {\Upsilon }\mathrm {Pb}$ contribution from the data.

## Extraction of cross sections

The dimuon events selected as described above are used to determine the differential $\mathrm {\Upsilon }$ photoproduction cross sections in four bins of $p_{\mathrm {T}} ^2$ over $p_{\mathrm {T}} ^2=0.01$–$1\,\text {GeV} {}^2$, and in four bins of *y* over $|y |<2.2$. Because of the limited size of the data sample, we first extract the differential cross sections for all $\mathrm {\Upsilon }\mathrm {(nS)}$ resonances combined. Then, the total cross section as a function of $W_{\gamma \mathrm {p}}$ is extracted for the $\mathrm {\Upsilon (1S)}$ state alone, as described below, and is compared with previous experimental measurements and theoretical predictions.

The background-subtracted $$p_{\mathrm {T}} ^2$$ and *y* distributions are first unfolded over the region $$0.01<p_{\mathrm {T}} ^2<1\,\text {GeV} {}^2$$, $$|y |<2.2$$, and muon $$p_{\mathrm {T}} ^{\mu }>3.3\,\text {GeV} $$, by using the Bayesian iterative unfolding technique [54], as implemented in the RooUnfold package [55], with four iterations. This procedure corrects for detector effects and data migration between bins. The response matrix is obtained from the starlight$$\gamma \mathrm {p} $$ simulation. The differential cross section $$\mathrm {d}\sigma /\mathrm {d}p_{\mathrm {T}} ^2$$ is further extrapolated to the full range of single-muon $$p_{\mathrm {T}} $$ by means of an acceptance correction factor $$A^{\text {corr}}=N_{\mathrm {\Upsilon }\mathrm {(nS)}}(p_{\mathrm {T}} ^{\mu }>3.3\,\text {GeV} $$$$)/N_{\mathrm {\Upsilon }\mathrm {(nS)}}(p_{\mathrm {T}} ^{\mu }>0)$$, estimated with the starlight$$\gamma \mathrm {p} $$ simulation. The measured $$\mathrm {d}\sigma /{\mathrm {d}}y$$ values in each rapidity bin are also similarly extrapolated down to zero dimuon $$p_{\mathrm {T}} $$. The $$A^{\text {corr}}\approx 0.6$$ factor does not significantly depend on $$p_{\mathrm {T}} ^2$$ but varies as a function of *y* as shown later in Table 3. The $$p_{\mathrm {T}} ^2$$- and *y*-differential cross sections, multiplied by the dimuon branching fraction, are extracted for the three $$\mathrm {\Upsilon }\mathrm {(nS)} $$ states combined as follows,

1 $$\begin{aligned} \begin{aligned} \sum _\mathrm {n}\mathcal {B}_{\mathrm {\Upsilon }\mathrm {(nS)} \rightarrow \mu ^+\mu ^-}\,\frac{\mathrm {d}\sigma _{\mathrm {\Upsilon }\mathrm {(nS)}}}{\mathrm {d}p_{\mathrm {T}} ^2}= & {} \frac{N^{\text {corr}}_{\mathrm {\Upsilon }\mathrm {(sum)}}}{\mathcal {L} \, \Delta p_{\mathrm {T}} ^2},\\ \sum _\mathrm {n}\mathcal {B}_{\mathrm {\Upsilon }\mathrm {(nS)} \rightarrow \mu ^+\mu ^-}\,\frac{\mathrm {d}\sigma _{\mathrm {\Upsilon }\mathrm {(nS)}}}{{\mathrm {d}}y}= & {} \frac{N^{\text {corr}}_{\mathrm {\Upsilon }\mathrm {(sum)}}}{\mathcal {L} \, \Delta y}. \end{aligned} \end{aligned}$$

Here $N^{\text {corr}}_{\mathrm {\Upsilon }\mathrm {(sum)}}$ denotes the background-subtracted, unfolded, and acceptance-corrected number of $\mathrm {\Upsilon (1S)}$, $\mathrm {\Upsilon (2S)}$ and $\mathrm {\Upsilon (3S)}$ signal events in each $p_{\mathrm {T}} ^2$ and *y* bin, $\mathcal {L}$ is the integrated luminosity, $\Delta p_{\mathrm {T}} ^2$ and $\Delta y$ are the widths of the $p_{\mathrm {T}} ^2$ and *y* bins, and $\mathcal {B}_{\mathrm {\Upsilon }\mathrm {(nS)} \rightarrow \mu ^+\mu ^-}$ is the dimuon branching fraction [53]. The differential $\mathrm {\Upsilon (1S)}$ photoproduction cross section $\mathrm {d}\sigma _{\mathrm {\Upsilon }{\mathrm {(1S)}}}/{\mathrm {d}}y$ is then extracted via

2 $$\begin{aligned} \frac{\mathrm {d}\sigma _{\mathrm {\Upsilon (1S)}}}{{\mathrm {d}}y}= & {} \frac{f_{\mathrm {\Upsilon (1S)}}}{\mathcal {B}_{\mathrm {\Upsilon (1S)}\rightarrow \mu ^+\mu ^-}(1+f_{\text {FD}})}\nonumber \\&\times \left[ \sum _\mathrm { n}\mathcal {B}_{\mathrm {\Upsilon }\mathrm {(nS)} \rightarrow \mu ^+\mu ^-}\frac{\mathrm {d}\sigma _{\mathrm {\Upsilon }\mathrm {(nS)}}}{{\mathrm {d}}y}\right] , \end{aligned}$$

where the factor $f_{\mathrm {\Upsilon (1S)}}$ is the ratio of $\mathrm {\Upsilon (1S)}$ to $\mathrm {\Upsilon }\mathrm {(sum)} =\mathrm {\Upsilon (1S)}+\mathrm {\Upsilon (2S)}+\mathrm {\Upsilon (3S)}$ events, $f_{\text {FD}}$ is the feed-down contribution to the $\mathrm {\Upsilon (1S)}$ events originating from the $\mathrm {\Upsilon (2S)}\rightarrow \mathrm {\Upsilon (1S)}+ X$ decays (where $X=\pi ^{+}\pi ^{-}$ or $\pi ^{0}\pi ^{0}$), and $\mathcal {B}_{\mathrm {\Upsilon (1S)}\rightarrow \mu ^+\mu ^-} = (2.48\pm 0.05)\%$ [53] is the branching fraction for the dimuon $\mathrm {\Upsilon (1S)}$ meson decay channel.

The fraction of $\mathrm {\Upsilon (1S)}$ to $\mathrm {\Upsilon }\mathrm {(sum)} =\mathrm {\Upsilon (1S)}+\mathrm {\Upsilon (2S)}+\mathrm {\Upsilon (3S)}$ yields is first derived from the event yield ratios $r_{21}=N_{\mathrm {\Upsilon (2S)}}/N_{\mathrm {\Upsilon (1S)}} = 0.78 \pm 0.31$ and $r_{31}=N_{\mathrm {\Upsilon (3S)}}/N_{\mathrm {\Upsilon (1S)}} = 0.21 \pm 0.22$ extracted from the invariant mass fit shown in Fig. 2, giving $f_{\mathrm {\Upsilon (1S)}}=(1+r_{21}+r_{31})^{-1} = 0.50 \pm 0.09$, where the correlation between the two fitted parameters was not taken into account. Since this fraction has a relatively large statistical uncertainty, we use the value derived from the analysis [51] of inclusive $\mathrm {\Upsilon }\mathrm {(nS)}$ meson production instead, which is performed at the same nucleon–nucleon collision centre-of-mass energy and in a similar $\mathrm {\Upsilon }$ rapidity range as the current $\mathrm {p}$Pb measurement, in which the fraction is expressed as a function of the number of additional charged particles in the event ($N_{\text {ch}}$) and extrapolated to $N_{\text {ch}}=0$. This procedure yields $f_{\mathrm {\Upsilon (1S)}}=0.68 \pm 0.04$, consistent within statistical uncertainties with the factor obtained from the current data, as well as with the $f_{\mathrm {\Upsilon (1S)}}=0.71 \pm 0.03$ and $0.73 \pm 0.05$ values obtained in the measurements based on proton-(anti)proton data by LHCb [34] and CDF [56], at very forward and central $\mathrm {\Upsilon }$ rapidities, respectively.

The feed-down contribution is estimated using the MC simulation in the following way: the initial $$\mathrm {\Upsilon (2S)}$$$$p_{\mathrm {T}} $$ and *y* distributions are taken from the starlight generator, and their $$\mathrm {\Upsilon (1S)}+ \pi \pi $$ decays, followed by $$\mathrm {\Upsilon (1S)}\rightarrow \mu ^{+}\mu ^{-}$$ are simulated with pythia  6.4 [57]. After applying all selections, the fraction of dimuon events from $$\mathrm {\Upsilon (2S)}$$ feed-down is found to be 8% of the exclusive signal $$\mathrm {\Upsilon (1S)}$$ events reconstructed using the starlight simulation. The contribution from feed-down of exclusive $$\chi _{\mathrm {b}} $$ states is neglected because these mesons can only be produced in double-pomeron exchange processes (or in pairs, via $$\gamma \gamma \rightarrow \chi _{\mathrm {b}} \chi _{\mathrm {b}} $$, with very small cross sections), which have comparatively much smaller yields in proton-nucleus collisions [58, 59].

Finally, the exclusive $\mathrm {\Upsilon (1S)}$ photoproduction cross section as a function of $W_{\gamma \mathrm {p}}$, is obtained from the $\mathrm {d}\sigma _{\mathrm {\Upsilon (1S)}}/{\mathrm {d}}y$ cross section via the relation

3 $$\begin{aligned} \sigma _{\gamma \mathrm {p}\rightarrow \mathrm {\Upsilon (1S)}\mathrm {p}}(W_{\gamma \mathrm {p}} ^{2}) = \frac{1}{\Phi }\frac{\mathrm {d}\sigma _{\mathrm {\Upsilon (1S)}}}{{\mathrm {d}}y}, \end{aligned}$$

in four different rapidity bins, with associated $W_{\gamma \mathrm {p}}$ intervals, given in Table 3. The cross sections are given at the value $W_{0}$, which corresponds to the average rapidity over a bin, $\langle y\rangle$. The photon flux $\Phi$ in Eq. (3), evaluated at $\langle y\rangle$, is obtained from the starlight simulation and calculated in the impact parameter space requiring the $\mathrm {p}$Pb separation to be larger than the sum of their radii.

## Systematic uncertainties

The following sources of systematic uncertainty are taken into account in the measurements of all differential and total $\mathrm {\Upsilon }$ meson production cross sections, as well as for the extraction of the exponential slope *b* of the $p_{\mathrm {T}} ^2$ spectrum:

- The muon reconstruction and selection efficiency has three components: the efficiency to find a track in the inner tracker, the efficiency to pass the track quality requirements, and the probability to pass the HLT selection. These efficiencies are estimated following the ”tag-and-probe” method [51], using first a sample of inclusive $\mathrm {\Upsilon (1S)}$ events selected with a trigger that requires two muons (to determine track and muon-quality efficiencies), and second a $\mathrm {\Upsilon (1S)}$ event sample similar to the one used in the nominal analysis, but collected with an independent trigger (to determine the trigger efficiency). The associated systematic uncertainty is evaluated from the difference in efficiencies obtained from the data and simulation, and it leads to uncertainties of $10.5\%$, $4.1\%$ and $1.7\%$ for track, muon-quality and trigger component, respectively. The overall uncertainty is estimated by adding the three numbers in quadrature, and leads to an $11\%$ uncertainty in the normalization of the cross sections, but no effect on the *b* slope measurement.
- To estimate the systematic uncertainty due to the model dependence of the acceptance correction, the parameters *b* and $\delta$ of the simulated starlight spectra are changed by ± 30% (chosen conservatively by the uncertainties of the corresponding fits to the data), and the resulting MC distributions are used for the determination of the extrapolation factor $A^\mathrm {corr}$, the unfolding, and the $\gamma \mathrm {Pb} \rightarrow \mathrm {\Upsilon }\mathrm {Pb}$ background subtraction, resulting in 2–3% changes in the measured observables.
- The uncertainty due to the unfolding procedure is studied by modifying the number of iterations used for the Bayesian unfolding from the nominal value of 4 to 3 and 5, resulting in an uncertainty of $1\%$ for the $p_{\mathrm {T}} ^2$ spectrum, $0.2\%$ for the *b* slope, and no change for the much flatter $\mathrm {d}\sigma /{\mathrm {d}}y$ distribution, which has negligible net bin-to-bin migrations.
- The uncertainty associated with the exclusive QED background contribution is estimated by comparing the starlight simulation to the data in sideband regions of the invariant mass distribution, $8.0<m_{\mu ^{+}\mu ^{-}}<9.1\,\text {GeV}$ and $10.6<m_{\mu ^{+}\mu ^{-}}<12.0\,\text {GeV}$, for $p_{\mathrm {T}} <0.15\,\text {GeV}$. The ratio of the simulation to the data in that region is found to be unity with a statistical uncertainty of 5%. To estimate the uncertainty due to the QED background subtraction, the MC normalization is scaled by ± 5%, resulting in 3–4% changes in the experimental observables.
- The uncertainty in the nonexclusive background contributions is estimated by varying the HF energy threshold by ± 10%. The resulting uncertainties of the observables vary between 3 and 6%.
- The uncertainty introduced by the $\mathrm {\Upsilon (2S)}\rightarrow \mathrm {\Upsilon (1S)}+X$ decays is estimated by modifying the values of the *b* and $\delta$ parameters of the $\mathrm {\Upsilon (2S)}$ spectra in the starlight MC to those obtained from the reweighting described in Sect. 4. This resulted in a ± 2% variation of the $\mathrm {\Upsilon (1S)}$ cross sections. The uncertainty in $f_{\mathrm {\Upsilon (1S)}}= \mathrm {\Upsilon (1S)}/\mathrm {\Upsilon }\mathrm {(sum)}$ is 7%, estimated as the quadratic sum of the uncertainty obtained from the extrapolation discussed in Sect. 5 and from the difference between this result and that obtained by LHCb in Ref. [34]. The latter takes into account possible differences between inclusive and exclusive processes in proton-proton and proton-lead collisions. An additional 2% uncertainty in the $\mathrm {\Upsilon (1S)}\rightarrow \mu ^+\mu ^-$ branching fraction is taken from the PDG world average [53]. All these uncertainties affect only the $\mathrm {\Upsilon (1S)}$ cross sections.
- The theoretical uncertainty in the photon flux affects only the total cross section $\sigma _{\gamma \mathrm {p} \rightarrow \mathrm {\Upsilon (1S)}\mathrm {p}}$ and is estimated by changing the Pb radius by ± 0.5$\text { fm}$, conservatively covering different estimates of the neutron skin thickness [60]. It amounts to 2, 3, 3, and 9% in the four *y* bins, respectively. The photon flux uncertainty (listed in the bottom row of Table 3) is larger for higher photon energies as they are dominated by smaller impact parameters.
- A systematic normalization uncertainty of ± 4% associated with the integrated luminosity [61] is assigned to the measurement of differential and total cross sections, with no effect on the *b* slope uncertainty.

**Table 1:** Relative systematic uncertainties in percent in the measurements of $\sum \mathcal {B}_{\mathrm {\Upsilon }\mathrm {(nS)} \rightarrow \mu ^+\mu ^-}\mathrm {d}\sigma /\mathrm {d}p_{\mathrm {T}} ^2$, the exponential *b* slope of the $p_{\mathrm {T}} ^2$ spectrum, $\sum \mathcal {B}_{\mathrm {\Upsilon }\mathrm {(nS)} \rightarrow \mu ^+\mu ^-}\mathrm {d}\sigma /{\mathrm {d}}y$, $\mathrm {d}\sigma _{\mathrm {\Upsilon (1S)}}/{\mathrm {d}}y$, and $\sigma _{\gamma \mathrm {p} \rightarrow \mathrm {\Upsilon (1S)}\mathrm {p}}$. Individual contributions, as well as total systematic uncertainties added in quadrature are presented. For the $p_{\mathrm {T}} ^2$- and *y*-differential cross sections, the values averaged over all bins are shown

Source	$\sum \mathcal {B}_{\mathrm {\Upsilon }\mathrm {(nS)} \rightarrow \mu ^+\mu ^-}\mathrm {d}\sigma /\mathrm {d}p_{\mathrm {T}} ^2$	*b*	$\sum \mathcal {B}_{\mathrm {\Upsilon }\mathrm {(nS)} \rightarrow \mu ^+\mu ^-}\mathrm {d}\sigma /{\mathrm {d}}y$	$\mathrm {d}\sigma _{\mathrm {\Upsilon (1S)}}/{\mathrm {d}}y$	$\sigma _{\gamma \mathrm {p} \rightarrow \mathrm {\Upsilon (1S)}\mathrm {p}}$
Muon efficiency	± 11	–	± 11	± 11	± 11
Acceptance	± 3	± 2	± 2	± 2	± 2
Unfolding	± 1	± 0.2	–	–	–
Exclusive QED background	± 4	± 3	± 4	± 4	± 4
Nonexclusive background	± 3	± 3	± 6	± 6	± 6
Integrated luminosity	± 4	–	± 4	± 4	± 4
Feed-down	–	–	–	± 2	± 2
Branching fraction $\mathcal {B}_{\mathrm {\Upsilon (1S)}}\rightarrow \mu ^+\mu ^-$	–	–	–	± 2	± 2
$f_{\mathrm {\Upsilon (1S)}}$ fraction	–	–	–	± 7	± 7
Photon flux $\Phi$	–	–	–	–	± 4
Total	± 13	± 5	± 14	± 16	± 16

The summary of the systematic uncertainties for all measurements is presented in Table 1. The dominant sources are the muon reconstruction efficiency and the modeling of the nonexclusive backgrounds. The total uncertainty is calculated by adding in quadrature the individual contributions, and varies between ± 5% for the *b* slope to ± 16% for $\sigma _{\gamma \mathrm {p} \rightarrow \mathrm {\Upsilon (1S)}\mathrm {p}}$. Given the limited integrated luminosity available, the measurements are dominated by statistical uncertainties.

## Results

### Differential cross section as a function of $p_{\mathrm {T}} ^2$ and *y*

**Fig. 4:**
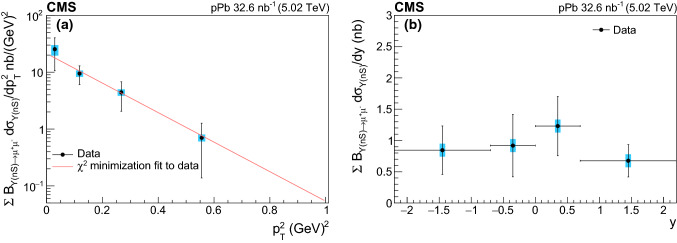
Differential $\mathrm {\Upsilon }\mathrm {(nS)} \rightarrow \mu ^+\mu ^-$ photoproduction cross section as a function of **a**$p_{\mathrm {T}} ^2$ and **b** rapidity *y*, measured in $\mathrm {p}$Pb collisions at $\sqrt{\smash [b]{s_{_{\mathrm {NN}}}}} = 5.02\,\text {TeV}$. In the left plot, the data points are placed along the abscissa following the prescription of [62], and the solid line is an exponential fit of the form $e^{-bp_{\mathrm {T}} ^2}$. In the right plot, the horizontal bars are shown to indicate the width of each *y* bin. In both plots, the vertical bars represent the statistical uncertainties and the boxes represent the systematic uncertainties

The differential cross sections (multiplied by the dimuon branching fractions) for exclusive $$\mathrm {\Upsilon }\mathrm {(nS)} $$ photoproduction, $$\sum \mathcal {B}_{\mathrm {\Upsilon }\mathrm {(nS)} \rightarrow \mu ^+\mu ^-}\mathrm {d}\sigma _{\mathrm {\Upsilon }\mathrm {(nS)}}/\mathrm {d}p_{\mathrm {T}} ^2$$ and $$\sum \mathcal {B}_{\mathrm {\Upsilon }\mathrm {(nS)} \rightarrow \mu ^+\mu ^-}\mathrm {d}\sigma _{\mathrm {\Upsilon }\mathrm {(nS)}}/{\mathrm {d}}y$$, measured over $$|y |<2.2$$, are shown in Fig. 4 and tabulated in Table  2. The $$p_{\mathrm {T}} ^2$$-differential cross section is fitted with an exponential function in the region $$0.01<p_{\mathrm {T}} ^2<1.0\,\text {GeV} $$$$^2$$, using a $$\chi ^2$$ goodness-of-fit minimization method. A slope of $$b=6.0 \pm 2.1 \,\text {(stat)} \pm 0.3 \,\text {(syst)} \,\text {GeV} ^{-2}$$ is extracted, in agreement with the value $$b=4.3^{+2.0}_{-1.3} \,\text {(stat)} {}^{+0.5}_{-0.6} \,\text {(syst)} \,\text {GeV} ^{-2}$$ measured by the ZEUS experiment [24] in the photon–proton centre-of-mass energy range $$60<W_{\gamma \mathrm {p}} <220\,\text {GeV} $$, and with the predictions of pQCD-based models [10].

**Table 2:** Differential exclusive $\mathrm {\Upsilon }\mathrm {(nS)} \rightarrow \mu ^+\mu ^-$ photoproduction cross sections in four $p_{\mathrm {T}} ^2$ and *y* bins. The first and second uncertainties correspond to statistical and systematic components, respectively

$p_{\mathrm {T}} ^2$ bin ($\text {GeV} ^2$)	$\sum \mathcal {B}_{\mathrm {\Upsilon }\mathrm {(nS)} \rightarrow \mu ^+\mu ^-}\,\mathrm {d}\sigma _{\mathrm {\Upsilon }\mathrm {(nS)}}/\mathrm {d}p_{\mathrm {T}} ^2$ (nb/$\text {GeV} ^2$)	*y* bin	$\sum \mathcal {B}_{\mathrm {\Upsilon }\mathrm {(nS)} \rightarrow \mu ^+\mu ^-}\,\mathrm {d}\sigma _{\mathrm {\Upsilon }\mathrm {(nS)}}/{\mathrm {d}}y$ (nb)
(0.01,0.05)	$25.4 \pm 14.8 \pm 4.9$	$(-\,2.2, -\,0.7)$	$0.8 \pm 0.4 \pm 0.1$
(0.05,0.20)	$9.5 \pm 3.4 \pm 1.1$	$(-\,0.7, 0.0)$	$0.9 \pm 0.5 \pm 0.1$
(0.20,0.35)	$4.4 \pm 2.4 \pm 0.5$	(0.0, 0.7)	$1.2 \pm 0.5 \pm 0.1$
(0.35,1.00)	$0.7 \pm 0.6 \pm 0.1$	(0.7, 2.2)	$0.7 \pm 0.2 \pm 0.1$

**Fig. 5:**
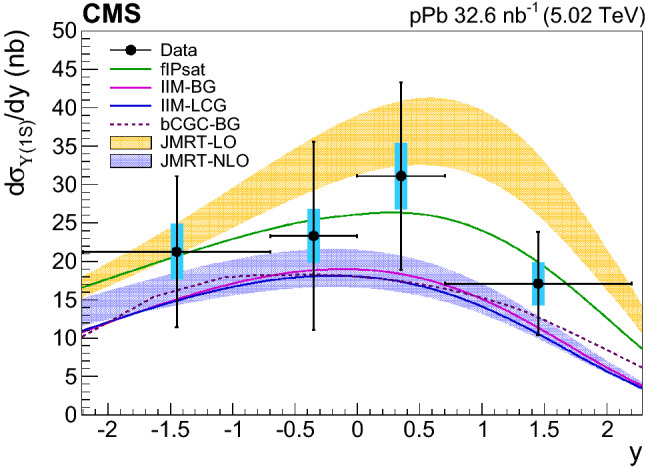
Differential $\mathrm {\Upsilon (1S)}$ photoproduction cross section as a function of rapidity measured in $\mathrm {p}$Pb collisions at $\sqrt{\smash [b]{s_{_{\mathrm {NN}}}}} = 5.02\,\text {TeV}$ in the dimuon rapidity region $|y |<2.2$, compared to various theoretical predictions [10, 15–19]. The horizontal bars are plotted to indicate the width of each *y* bin. The vertical bars represent the statistical uncertainties and the boxes represent the systematic uncertainties

Figure 5 shows the rapidity distribution of the $\mathrm {\Upsilon (1S)}$ state obtained according to Eq. (2). The values of all relevant parameters needed to compute the $\mathrm {\Upsilon (1S)}$ cross sections in the four rapidity bins under consideration are listed in Table  3. The CMS measurements are compared to the following theoretical predictions:

- The JMRT model [10], a pQCD approach that uses standard (collinear) PDFs with a skewness factor to approximate GPDs, including LO and NLO corrections, and a gap survival factor to account for the exclusive production;
- The factorized impact parameter saturation model, fIPsat, with an eikonalized gluon distribution function that uses the colour glass condensate (CGC) formalism to incorporate gluon saturation at low *x* [17, 18];
- the Iancu, Itakura and Munier (IIM) colour dipole formalism [63] with two sets of meson wave functions, boosted Gaussian (BG) and light-cone Gaussian (LCG), which also incorporate saturation effects [15, 16];
- the impact parameter CGC model (bCGC), which takes into account the *t*-dependence of the differential cross section, using the BG wave function [19, 64].

As shown in Fig. 5, most theoretical predictions are consistent with the data, within the relatively large experimental uncertainties, with the JMRT-LO results being systematically above the data points as well as all the other calculations.

**Table 3:** Values of the $\mathrm {\Upsilon (1S)}$ photoproduction cross section in four rapidity *y* bins, corresponding to four photon–proton $W_{\gamma \mathrm {p}}$ centre-of-mass energy ranges (with central $W_{0}$ value obtained following the procedure outlined in Ref. [62]), in $\mathrm {p}$Pb collisions at $\sqrt{\smash [b]{s_{_{\mathrm {NN}}}}} = 5.02\,\text {TeV}$. The symbols $N_{\mathrm {\Upsilon }\mathrm {(sum)}}^{\text {back-sub}}$, $N_{\mathrm {\Upsilon }\mathrm {(sum)}}^{\text {unfol}}$, and $N_{\mathrm {\Upsilon }\mathrm {(sum)}}^{\text {corr}}$ represent the numbers of $\mathrm {\Upsilon }\mathrm {(sum)} =\mathrm {\Upsilon (1S)}+\mathrm {\Upsilon (2S)}+\mathrm {\Upsilon (3S)}$ candidates after background subtraction, unfolding, and extrapolation with the $A^{\text {corr}}$ factor, respectively; $N_{\mathrm {\Upsilon (1S)}}$ is the extracted number of $\mathrm {\Upsilon (1S)}$ mesons, and $\Phi$ is the theoretical effective photon flux (see text). The first (second, if given) uncertainty quoted corresponds to the statistical (systematic) component

*y* range	$(-\,2.2,-\,0.7)$	$(-\,0.7,0.0)$	(0.0, 0.7)	(0.7, 2.2)
$\langle y\rangle$	$-1.45$	$-0.35$	0.35	1.45
$N_{\mathrm {\Upsilon }\mathrm {(sum)}}^{\text {back-sub}}$	$14 \pm 6$	$9 \pm 5$	$12 \pm 5$	$12 \pm 5$
$N_{\mathrm {\Upsilon }\mathrm {(sum)}}^{\text {unfol}}$	$19 \pm 9$	$13 \pm 7$	$17\pm 7$	$16 \pm 6$
$A^{\text {corr}}$	$0.46 \pm 0.01$	$0.61 \pm 0.01$	$0.61 \pm 0.01$	$0.50 \pm 0.01$
$N_{\mathrm {\Upsilon }\mathrm {(sum)}}^{\text {corr}}$	$41 \pm 19 \pm 7$	$21 \pm 11 \pm 3$	$28 \pm 11 \pm 4$	$33 \pm 13 \pm 5$
$N_{\mathrm {\Upsilon (1S)}}=\frac{f_{\mathrm {\Upsilon (1S)}}N_{\mathrm {\Upsilon }\mathrm {(sum)}}}{(1+f_{\mathrm {FD})}}$	$26 \pm 12 \pm 4$	$13 \pm 7 \pm 2$	$18 \pm 7 \pm 2$	$21 \pm 8 \pm 3$
$\mathrm {d}\sigma _{\mathrm {\Upsilon (1S)}}/{\mathrm {d}}y$ (nb)	$21 \pm 10 \pm 4$	$23 \pm 12 \pm 3$	$31 \pm 12 \pm 4$	$17 \pm 7 \pm 3$
$W_{\gamma \mathrm {p}}$ range ($\text {GeV}$)	91–194	194–275	275–390	390–826
$W_{0}$ ($\text {GeV}$)	133	231	328	568
Photon flux ($\Phi$)	$102.2 \pm 2.0$	$68.3 \pm 2.0$	$46.9 \pm 1.4$	$17.9 \pm 1.6$
$\sigma _{\gamma \mathrm {p} \rightarrow \mathrm {\Upsilon (1S)}\mathrm {p}}$ (pb)	$208\pm 96 \pm 37$	$343\pm 180 \pm 51$	$663 \pm 260 \pm 93$	$956 \pm 376 \pm 162$

### Cross section as a function of $W_{\gamma \mathrm {p}}$

**Fig. 6:**
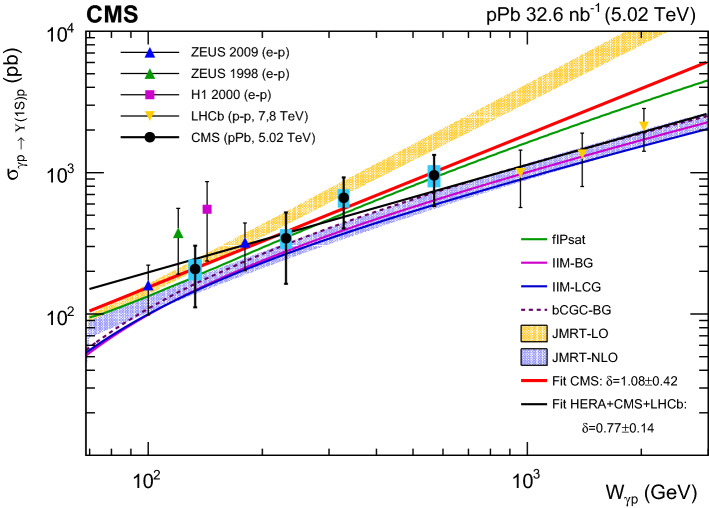
Cross section for exclusive $\mathrm {\Upsilon (1S)}$ photoproduction, $\gamma \mathrm {p} \rightarrow \mathrm {\Upsilon (1S)}\mathrm {p}$, as a function of photon–proton centre-of-mass energy, $W_{\gamma \mathrm {p}}$, compared to previous HERA [20–22] and LHCb [34] data as well as to various theoretical predictions [10, 15–19]. The vertical bars represent the statistical uncertainties and the boxes represent the systematic uncertainties

The values of the $\sigma _{\gamma \mathrm {p} \rightarrow \mathrm {\Upsilon (1S)}\mathrm {p}}$ cross section obtained via Eq. (3) are plotted as a function of $W_{\gamma \mathrm {p}}$ in Fig. 6, together with the previous measurements from H1 [20], ZEUS [21, 22], and LHCb [34], and the five model predictions described in the previous section. The CMS results (listed in Table 3) cover the range of energies between the HERA and LHCb data. As $\sigma (W_{\gamma \mathrm {p}})$ is, to first approximation, proportional to the square of the gluon density of the proton, and since the gluon distribution at low Bjorken *x* is well described by a power law, the cross section also follows a power-law energy dependence. A fit of the extracted CMS $\sigma _{\gamma \mathrm {p} \rightarrow \mathrm {\Upsilon (1S)}\mathrm {p}}$ cross section with a function of the form $A\,(W_{\gamma \mathrm {p}} [\text {GeV} ]/400)^{\delta }$ (with the constant *A* corresponding to the cross section at the middle value, $W_{\gamma \mathrm {p}} = 400\,\text {GeV}$, over the range of energies covered) gives $\delta =1.08\pm 0.42$ and $A=690\pm 183\text { pb}$ (black solid line in Fig 6), consistent with the value $\delta =1.2 \pm 0.8$ obtained by ZEUS [21]. A similar fit to the CMS, H1 [20], and ZEUS [21] data together gives $\delta =0.99\pm 0.27$, in good agreement with the results of the fit to the CMS data alone. The fit over the whole kinematic range, including the higher-$W_{\gamma \mathrm {p}}$ LHCb data, yields an exponent of $\delta =0.77\pm 0.14$, consistent with the collision-energy dependence of the $\mathrm {J}/\psi$ photoproduction and light vector meson electroproduction cross sections [65].

The data are compared to the predictions of the JMRT model, including LO and NLO corrections. A fit with the power-law function in the entire $W_{\gamma \mathrm {p}}$ range of the data yields $\delta =1.39$ and $\delta =0.84$ for the LO and NLO calculations, respectively. The LO predictions show a steeper increase of the cross section with energy than seen in the data over the full kinematic range. The NLO prediction reproduces the measured rise of the cross section with $W_{\gamma \mathrm {p}}$. The recent LHCb results at higher $W_{\gamma \mathrm {p}}$ [34] also disfavour the JMRT LO prediction. Figure 6 shows theoretical predictions from the fIPsat, IIM, and bCGC models, which overall bracket the combined HERA and LHC results. The fIPsat calculations are consistent with the CMS measurement, but predict a somewhat higher cross section than that measured by LHCb. The IIM and bCGC predictions satisfactorily describe the rise of the cross section with $\gamma \mathrm {p}$ centre-of-mass energy. As discussed in Ref. [10], the gluon PDF associated with the JMRT NLO prediction, which is consistent with the CMS+LHCb data presented here, has a somewhat different shape at low-*x* than that predicted by standard pQCD collinear fits used at the LHC such as CT14 [66], NNPDF3.0 [67], and MMHT [68]. However, given the currently large statistical uncertainty of the results presented here, an improved understanding of the low-*x* gluon density, and its evolution with energy scale, will require more precise measurements with larger integrated luminosities and/or at higher centre-of-mass energies.

## Summary

The first study of the exclusive photoproduction of $\mathrm {\Upsilon }$(1S,2S,3S) mesons, in the $\mu ^{+}\mu ^{-}$ decay mode, from protons in ultraperipheral $\mathrm {p}$Pb collisions at $\sqrt{\smash [b]{s_{_{\mathrm {NN}}}}} = 5.02\,\text {TeV}$, has been reported using data collected with the CMS detector corresponding to an integrated luminosity of 32.6$\,\text {nb}^{-1}$. The differential cross section $\mathrm {d}\sigma /\mathrm {d}p_{\mathrm {T}} ^2$ and associated exponential slope *b* have been measured in the squared transverse momentum range $p_{\mathrm {T}} ^2<1.0\,\text {GeV} ^2$. The extracted value of $b=6.0 \pm 2.1 \,\text {(stat)} \pm 0.3 \,\text {(syst)} \,\text {GeV} ^{-2}$ is consistent with the slope measurement at other centre-of-mass energies. The exclusive $\mathrm {\Upsilon (1S)}$ photoproduction cross sections, differential in rapidity *y* and as a function of the photon–proton centre-of-mass energy $W_{\gamma \mathrm {p}}$, have been measured in the range $91< W_{\gamma \mathrm {p}} < 826\,\text {GeV}$. Such measurements probe the region of parton fractional momenta $x\approx 10^{-4}$–$10^{-2}$ in the proton, bridging a previously unexplored region between the HERA and LHCb measurements. The dependence of $\sigma _{\gamma \mathrm {p} \rightarrow \mathrm {\Upsilon (1S)}\mathrm {p}}$ on $W_{\gamma \mathrm {p}}$ is well described by a power law with an exponent smaller than that predicted by leading order perturbative quantum chromodynamics (pQCD) approaches. The exponent is, however, consistent with that extracted from a fit to the HERA and LHCb data, and with that predicted by next-to-leading-order pQCD calculations. The data, within their currently large statistical uncertainties, are consistent with various pQCD approaches that model the behaviour of the low-*x* gluon density, and provide new insights on the gluon distribution in the proton in this poorly explored region.

## Data Availability

This manuscript has no associated data or the data will not be deposited. [Authors’ comment: Release and preservation of data used by the CMS Collaboration as the basis for publications is guided by the CMS policy as written in its document “CMS data preservation, re-use and open access policy” (https://cms-docdb.cern.ch/cgibin/PublicDocDB/RetrieveFile?docid=6032&filename=CMSDataPolicyV1.2.pdf&version=2).]

## References

[1] Baltz AJ (2008). The physics of ultraperipheral collisions at the LHC. Phys. Rep..

[2] Klein S, Nystrand J (1999). Exclusive vector meson production in relativistic heavy ion collisions. Phys. Rev. C.

[3] d’Enterria D (2008). Quarkonia photoproduction at nucleus colliders. Nucl. Phys. B Proc. Suppl..

[4] Budnev V, Ginzburg IF, Meledin GV, Serbo VG (1975). The two photon particle production mechanism. Physical problems, applications, equivalent photon approximation. Phys. Rep..

[5] Lansberg JP (2008). Perspectives on heavy-quarkonium production at the LHC. AIP Conf. Proc..

[6] Brodsky SJ (1994). Diffractive leptoproduction of vector mesons in QCD. Phys. Rev. D.

[7] Frankfurt LL, Freund A, Strikman M (1998). Diffractive exclusive photoproduction in DIS at HERA. Phys. Rev. D.

[8] Frankfurt L, McDermott M, Strikman M (2001). A fresh look at diffractive $${\text{J}/\psi }$$ photoproduction at HERA, with predictions for THERA. JHEP.

[9] Martin AD, Nockles C, Ryskin MG, Teubner T (2008). Small $$x$$ gluon from exclusive $${\text{ J }/\psi }$$ production. Phys. Lett. B.

[10] Jones SP, Martin AD, Ryskin MG, Teubner T (2013). Probes of the small $$x$$ gluon via exclusive $${\text{ J }/\psi }$$ and $$\Upsilon $$ production at HERA and LHC. JHEP.

[11] Adeluyi A, Bertulani CA, Murray MJ (2012). Nuclear effects in photoproduction of heavy quarks and vector mesons in ultraperipheral PbPb and pPb collisions at the LHC. Phys. Rev. C.

[12] Adeluyi A, Nguyen T (2013). Coherent photoproduction of $$\psi $$ and $$\Upsilon $$ mesons in ultraperipheral pPb and PbPb collsions at the CERN LHC. Phys. Rev. C.

[13] Guzey V, Zhalov M (2013). Exclusive $${\text{ J }/\psi }$$ production in ultraperipheral collisions at the LHC: constraints on the gluon distributions in the proton and nuclei. JHEP.

[14] Guzey V, Kryshen E, Strikman M, Zhalov M (2013). Evidence for nuclear gluon shadowing from the ALICE measurements of PbPb ultraperipheral exclusive $${\text{ J }/\psi }$$ production. Phys. Lett. B.

[15] Sampaio dos Santos G, Machado MVT (2014). Exclusive photoproduction of quarkonium in proton-nucleus collisions at the CERN Large Hadron Collider. Phys. Rev. C.

[16] Sampaio dos Santos G, Machado MVT (2015). On theoretical uncertainty of color dipole phenomenology in the $${\text{ J }/\psi }$$ and $$\Upsilon $$ photoproduction in pA and AA collisions at the CERN Large Hadron Collider. J. Phys. G.

[17] Lappi T, Mantysaari H (2011). Incoherent diffractive $${\text{ J }/\psi }$$ production in high energy nuclear DIS. Phys. Rev. C.

[18] Lappi T, Mantysaari H (2013). $${\text{ J }/\psi }$$ production in ultraperipheral Pb+Pb and p+Pb collisions at energies available at the CERN large Hadron collider. Phys. Rev. C.

[19] Goncalves VP, Moreira BD, Navarra FS (2017). Exclusive heavy vector meson photoproduction in hadronic collisions at LHC: predictions of the color glass condensate model for run 2 energies. Phys. Rev. D.

[20] H1 Collaboration, Elastic photoproduction of $${\text{ J }/\psi }$$ and $$\Upsilon $$ mesons at HERA. Phys. Lett. B **483**, 23 (2000). 10.1016/S0370-2693(00)00530-X. arXiv:hep-ex/0003020

[21] ZEUS Collaboration, Exclusive photoproduction of $$\Upsilon $$ mesons at HERA. Phys. Lett. B **680**, 4 (2009). 10.1016/j.physletb.2009.07.066. arXiv:0903.4205

[22] ZEUS Collaboration, Measurement of elastic $$\Upsilon $$ photoproduction at HERA. Phys. Lett. B **437**, 432 (1998). 10.1016/S0370-2693(98)01081-8. arXiv:hep-ex/9807020

[23] H1 Collaboration, Elastic and proton-dissociative photoproduction of $${\text{ J }/\psi }$$ mesons at HERA. Eur. Phys. J. C **73**, 2466 (2013). 10.1140/epjc/s10052-013-2466-y. arXiv:1304.5162

[24] ZEUS Collaboration, Measurement of the $$t$$ dependence in exclusive photoproduction of $$\Upsilon \text{(1S) }$$ mesons at HERA. Phys. Lett. B **708**, 14 (2012). 10.1016/j.physletb.2012.01.009. arXiv:1111.2133

[25] Brodsky SJ (1999). The QCD pomeron with optimal renormalization. JETP.

[26] LHCb Collaboration, Exclusive $${\text{ J }/\psi }$$ and $$\psi $$(2S) production in pp collisions at $$\sqrt{s}=7$$ TeV. J. Phys. G **40**, 045001 (2013). 10.1088/0954-3899/40/4/045001. arXiv:1301.7084

[27] LHCb Collaboration, Updated measurements of exclusive $${\text{ J }/\psi }$$ and $$\psi $$(2S) production cross-sections in pp collisions at $$\sqrt{s}=7$$ TeV. J. Phys. G. **41**, 055002 (2014). 10.1088/0954-3899/41/5/055002. arXiv:1401.3288

[28] LHCb Collaboration, Central exclusive production of $${\text{ J }/\psi }$$ and $$\psi $$(2S) mesons in pp collisions at $$\sqrt{s}=13$$ TeV. JHEP. **10**, 167 (2018). 10.1007/JHEP10(2018)167. arXiv:1806.04079

[29] CMS Collaboration, Coherent $${\text{ J }/\psi }$$ photoproduction in ultra-peripheral PbPb collisions $$\sqrt{{s_{{\rm NN}}}} = 5.02 \text{ TeV } = 2.76 \text{ TeV }$$ with the CMS experiment. Phys. Lett. B ** 772**, 489 (2017). 10.1016/j.physletb.2017.07.001. arXiv:1605.06966

[30] ALICE Collaboration, Coherent $${\text{ J }/\psi }$$ photoproduction in ultra-peripheral PbPb collisions at $$\sqrt{{s_{_{\rm NN}}}} = 5.02 \text{ TeV } = 2.76 \text{ TeV }$$. Phys. Lett. B, **718**, 1273 (2013). 10.1016/j.physletb.2012.11.059. arXiv:1209.3715

[31] ALICE Collaboration, Charmonium and $$e^+e^-$$ pair photoproduction at mid-rapidity in ultra-peripheral PbPb collisions at $$\sqrt{{s_{{\rm NN}}}} = 5.02 \text{ TeV } = 2.76 \text{ TeV }$$. Eur. Phys. J. C, ** 73**, 2617 (2013). 10.1140/epjc/s10052-013-2617-1. arXiv:1305.1467 10.1140/epjc/s10052-013-2617-1 PMC4371050 25814847

[32] ALICE Collaboration, Exclusive $${\text{ J }/\psi }$$ photoproduction off protons in ultra-peripheral pPb collisions at $$\sqrt{{s_{{\rm NN}}}} = 5.02 \text{ TeV } = 5.02 \text{ TeV }$$. Phys. Rev. Lett. ** 113**, 232504 (2014). 10.1103/PhysRevLett.113.232504. arXiv:1406.7819 10.1103/PhysRevLett.113.232504 25526123

[33] ALICE Collaboration, Energy dependence of exclusive $${\text{ J }/\psi }$$ photoproduction off protons in ultra-peripheral pPb collisions at $$\sqrt{{s_{{\rm NN}}}} = 5.02 \text{ TeV } = 5.02 \text{ TeV }$$. (2018). arXiv:1809.03235

[34] LHCb Collaboration, Measurement of the exclusive $$\Upsilon $$ production cross section in pp collisions at $$\sqrt{s}=7$$ TeV and 8 TeV. * JHEP*, ** 09**, 084 (2015). 10.1007/JHEP09(2015)084. arXiv:1505.08139

[35] Jones SP, Martin AD, Ryskin MG, Teubner T (2016). Exclusive $${\text{ J }/\psi }$$ and $$\Upsilon $$ photoproduction and the low $$x$$ gluon. J. Phys. G.

[36] Gribov LV, Levin EM, Ryskin MG (1983). Semihard processes in QCD. Phys. Rep..

[37] Mueller AH, Qiu J-W (1986). Gluon recombination and shadowing at small values of $$x$$. Nucl. Phys. B.

[38] McLerran LD, Venugopalan R (1994). Gluon distribution functions for very large nuclei at small transverse momentum. Phys. Rev. D.

[39] CMS Collaboration, Description and performance of track and primary-vertex reconstruction with the CMS tracker. JINST **9**, P10009 (2014). 10.1088/1748-0221/9/10/P10009. arXiv:1405.6569

[40] CMS Collaboration, Performance of CMS muon reconstruction in pp collision events at $$\sqrt{s}=7$$ TeV. JINST **7**, P10002 (2012). 10.1088/1748-0221/7/10/P10002. arXiv:1206.4071

[41] CMS Collaboration, Status of zero degree calorimeter for CMS experiment. AIP Conf. Proc. **867**, 258 (2006). 10.1063/1.2396962. arXiv:nucl-ex/0608052

[42] CMS Collaboration, The CMS trigger system. JINST **12**, P01020 (2017). 10.1088/1748-0221/12/01/P01020. arXiv:1609.02366

[43] CMS Collaboration, The CMS experiment at the CERN LHC. JINST **3**, S08004 (2008). 10.1088/1748-0221/3/08/S08004

[44] Klein SR, Nystrand J (2004). Photoproduction of quarkonium in proton-proton and nucleus-nucleus collisions. Phys. Rev. Lett..

[45] J. Nystrand, Photons and exclusive processes at hadron colliders. Proceedings of PHOTON2009, Hamburg, Germany, **286** (2009). 10.3204/DESY-PROC-2009-03/Nystrand, arXiv:1001.4746

[46] Weizsäcker CFV (1934). Radiation emitted in collisions of very fast electrons. Z. Phys..

[47] Williams EJ (1934). Nature of the high-energy particles of penetrating radiation and status of ionization and radiation formulae. Phys. Rev..

[48] GEANT4 Collaboration, Geant4–a simulation toolkit. Nucl. Instrum. Meth. A **506**, 250 (2003). 10.1016/S0168-9002(03)01368-8

[49] Allison J (2006). Geant4 developments and applications. IEEE Trans. Nucl. Sci..

[50] Allison J (2016). Recent developments in Geant4. Nucl. Instrum. Methods A.

[51] CMS Collaboration, Event activity dependence of $$\Upsilon $$(nS) production in $$\sqrt{{s_{{\rm NN}}}} = 5.02 \text{ TeV }$$ pPb and $$\sqrt{s}= 2.76$$ TeV pp collisions. JHEP ** 04**, 103 (2014). 10.1007/JHEP04(2014)103, arXiv:1312.6300

[52] W. Verkerke, D. P. Kirkby, The RooFit toolkit for data modeling, in *13th International Conference for Computing in High-Energy and Nuclear Physics (CHEP 2003)*, p. MOLT007. SLAC, La Jolla, CA, US, March, 2003. arXiv:physics/0306116. eConf:C0303241/MOLT007

[53] Particle Data Group, Review of particle physics. Chin. Phys. C **40**, 100001 (2016). 10.1008/1674-1137/40/10/100001

[54] D’Agostini G (1995). A multidimensional unfolding method based on Bayes’ theorem. Nucl. Instrum. Methods A.

[55] T. Adye, Unfolding algorithms and tests using RooUnfold, in H. Prosper and L. Lyons, (eds.) *PHYSTAT 2011 Workshop on Statistical Issues Related to Discovery Claims in Search Experiments and Unfolding*, p. 313. Geneva, Switzerland, 2011. 10.5170/CERN-2011-006.313. arXiv:1105.1160

[56] CDF Collaboration, $$\Upsilon $$ production and polarization in $${\text{ p }}\bar{\text{ p }}$$ collisions at $$\sqrt{s}=$$ 1.8 TeV. Phys. Rev. Lett. **88**, 161802 (2002) 10.1103/PhysRevLett.88.161802

[57] Sjöstrand T, Mrenna S, Skands P (2006). PYTHIA 6.4 physics and manual. JHEP.

[58] Schramm AJ, Reeves DH (1997). Production of $$\eta $$ mesons in double pomeron exchange. Phys. Rev. D.

[59] Harland-Lang LA, Khoze VA, Ryskin MG, Stirling WJ (2010). Standard candle central exclusive processes at the Tevatron and LHC. Eur. Phys. J. C.

[60] Loizides C, Kamin J, d’Enterria D (2018). Improved Monte Carlo Glauber predictions at present and future nuclear colliders. Phys. Rev. C.

[61] CMS Collaboration, Luminosity calibration for the 2013 proton-lead and proton-proton data taking, CMS Physics Analysis Summary CMS-PAS-LUM-13-002 (2013)

[62] Lafferty GD, Wyatt TR (1995). Where to stick your data points: the treatment of measurements within wide bins. Nucl. Instrum. Methods A.

[63] Iancu E, Itakura K, Munier S (2004). Saturation and BFKL dynamics in the HERA data at small x. Phys. Lett. B.

[64] Goncalves VP (2017). Color dipole predictions for the exclusive vector meson photoproduction in pp/pPb/PbPb collisions at Run 2 LHC energies. Phys. Rev. D.

[65] Favart L, Guidal M, Horn T, Kroll P (2016). Deeply virtual meson production on the nucleon. Eur. Phys. J. A.

[66] Dulat S (2016). New parton distribution functions from a global analysis of quantum chromodynamics. Phys. Rev. D.

[67] NNPDF Collaboration, Parton distributions for the LHC Run II. * JHEP*** 04**, 040 (2015). 10.1007/JHEP04(2015)040. arXiv:1410.8849

[68] Harland-Lang LA, Martin AD, Motylinski P, Thorne RS (2015). Parton distributions in the LHC era: MMHT 2014 PDFs. Eur. Phys. J. C.

